# Why do Some Respond and Develop more from Coaching than Others? A Systematic Review of Coachability and Its Constituent Components

**DOI:** 10.1007/s40279-025-02267-6

**Published:** 2025-07-12

**Authors:** Stephen MacGabhann, Phillip Newman, Jeremy Witchalls, Rebecca Dowse, Gordon Waddington, Stephen Cobley

**Affiliations:** 1 Faculty of Health, Research Institute for Sport and Exercise, University of Canberra, Canberra, ACT Australia; 2 Discipline of Exercise and Sport Science, School of Health Sciences, Faculty of Medicine and Health, The University of Sydney, Susan Wakil Health Building, Sydney, NSW 2006 Australia; 3 New South Wales Institute of Sport (NSWIS), Sydney, NSW Australia

## Abstract

**Background:**

As an inter-individual psychological and behavioural construct, coachability is hypothesised to be a fundamental determinant of learning and performance enhancement within sport, business and education domains. Despite its perceived importance, consensus on a precise definition and its exact dimensional components has yet to be established.

**Objectives:**

This systematic review aimed to: (1) establish consensus on a common definition and (2) identify consistent constituent component constructs of coachability evident in the literature.

**Methods:**

Using systematic search and screening methods, 53 articles (41 studies; 12 grey literature) from sources spanning 5 decades were identified.

**Results:**

Findings revealed variable definitions of coachability that evolved with respective theoretical and investigatory paradigm shifts. Nonetheless, throughout, coachability was consistently considered a multi-component construct. Literature supported the existence of potentially six inter-connected component dimensions: *Attentiveness to information*; *Willingness to learn*; *Persistence in overcoming setbacks*; *Feedback seeking*; *Feedback receptivity*; and *Feedback implementation.* For each component, evidence illustrating psychological and behavioural features resonating with higher and lower component presence was identified. Identified components were also validated through independent interviews with sport coaches. Collectively, findings established a synthesised consensus definition aligned with identified component dimensions, reflecting: *an individual’s willingness and ability to seek, receive and act upon constructive feedback to persistently foster self-development and enhance performance in a given domain*.

**Conclusions:**

Given the limited assessments, methodologies and instruments available, research is recommended to refine and validate assessment instruments, verify constituent components identified, confirm relationships between coachability and developmental outcomes, and identify coachability optimisation strategies to benefit personal development within domain contexts.

## Key Points

**Table:** 

Coachability is defined as an individual’s willingness and ability to seek, receive and act upon constructive feedback to foster self-development and enhance performance across sport, business and educational domains.
Six consistent constituent components (dimensions) of coachability were identified: Attentiveness to Information, Willingness to Learn, Persistence in Overcoming Setbacks, Feedback Seeking, Feedback Receptivity and Feedback Implementation.
As a multi-component construct, coachability has potential explanatory value in accounting for cognitive and behavioural individual differences associated with (sub-)optimal learning and development outcomes across multiple domains.
While coachability can be assessed, future research is recommended to develop standardised multi-dimensional assessment instruments to validate coachability components and explore its optimisation for learning and performance outcomes.

## Introduction

### A Brief Historical Background

In their seminal text “Problem Athletes and How to Handle Them”, Ogilvie and Tutko [1] introduced ‘coachability’ to the vernacular of the sport psychology and coaching disciplines. Originally characterised as an individual difference trait, encompassing a blend of personality traits, cognitive responses and motivation-driven behaviours, Ogilvie and Tutko [1] proposed coachability as a psychological construct capable of elucidating and interpreting observable variations in athletic behaviour and accomplishment: “One of the most critical qualities for achieving greatness in athletic pursuits. Those who are deemed great professionals, with only a few exceptions, consistently exhibit high levels of coachability.” At the centre of their argument was the contention that less successful athletes failed to acquire or consistently demonstrate desirable coachability owing to the presence of three types of psycho-social barriers. These ranged from acute emotions to more enduring sub-conscious factors, including forms of psychological inhibition, psycho-social problems or pathological conditions, which subsequently impeded athletic cognitive and behavioural engagement.

Despite Ogilvie and Tutko’s introduction, possibly because of being considered as more a personality-driven individual character difference [2], coachability received limited immediate attention from researchers, although practical sports coaching interest was somewhat more pervasive [3, 4]. Likely connected to changes in psychological frameworks, expanding research understanding and a combination of societal and technology/industry organisational changes, research interest began to increase in the late 1990s to early 2000s. Importantly, coachability came to be viewed as a set of acquired competencies. Competencies were identified as being observable behavioural actions, with an underlying assumption of being malleable or developable [5–7]. Further, the absence, frequency or extent of competency presence could be potentially measured, reflecting underlying psychological functioning, including cognitive beliefs or attitudes as well as attentional, motivational and emotional control capabilities. Capability within each underlying competency was subsequently viewed as contributing toward an individual’s responsiveness to coaching instructions or social exchanges.

### How has Coachability Been Understood in Different Contexts?

Aligned with the competency view and summarising sport psychology-related literature in the last 20 years, coachability has been described as an individual difference construct, with possibly multi-dimensional components, including a general positive attitude and openness toward receiving instruction [8, 9], being accepting and integrating feedback [10], being engaged, committed and willing in the learning process [9, 11], actively seeking, receptive and utilising feedback [11–13], as well as demonstrating the implementation of coaching advice/instruction to improve personal performance [9, 11, 14]. However, across and within related studies, coachability and underlying competencies were either examined exploratorily, were not the primary focus of investigation, were not consistently assessed using similar or standardised methodologies, and assessment was frequently based on coaches’ perceptions of personality traits associated with successful athletic performance [8, 10, 15].

Given the potential relevance of underlying coachability competencies to learning and performance, it is perhaps unsurprising that business and industry-related research interests have expanded. Within business management coaching, [16] it was suggested that individuals could be evaluated on levels from being ‘un-coachable’ (psychologically affected) to ‘highly coachable’ (responsible, self-directed, showing a desire to improve, being lifelong learners) with corresponding characteristics. Subsequently, several similar coachability-associated competencies seem to have been emphasised (e.g. willingness and capability to seek, receive and act upon feedback to enhance performance) as demonstrating high coachability. These competencies have been identified as having positive relationships with performance in a variety of settings, including employee coachability [17], employee commitment and pro-organisational behaviour [18], entrepreneurship [19, 20], investor decision making [21, 22], leadership effectiveness [23] and sales performance [24].

### Why is Interest in Coachability Growing?

In sport, re-emerging interest in coachability has likely grown hand-in-hand with increasing commercialisation and professionalised performance expectations [25–27]. As a result, there is implicit interest in understanding the factors leading to high achievement and the ability to identify individuals who demonstrate high responsiveness to such demands [28, 29]. Similarly, understanding the individual factors and processes (e.g. coaching relationships) that contribute to maintaining overall mental health and well-being in highly demanding sports contexts has gained significance [30–32]. Within athlete development research and practice, there is demand to not only systematically develop both specific sport task-related physical and physiological capacities, but also a wide range of biomechanical, cognitive and psychological skills that underpin performance within the time frames available for such programmes [33, 34]. Consequently, if athletes do not exhibit the characteristic behaviours or competencies associated with coachability, or are not targeted for development, then individual performance progression may be sub-optimal or compromised, leading to potential performance, selection or contract loss for the athlete, alongside financial costs and resource allocation inefficiencies for the organisation [35, 36].

Within business and educational settings, changes in technology, organisational and working practices have also led to the necessity for leaders, entrepreneurs and employees to be adaptable and productive in dynamic environments [37, 38]. As a result, an increased emphasis on personal and professional skill development to meet evolving technology, market trends and job task demands has occurred [39, 40]. Thus, at an individual level, in recruitment, performance evaluations and training programme situations and cognitive and behavioural capabilities to adapt, be open to feedback, show a willingness to learn and demonstrate responsiveness have become highly valued. Consequently, the literature in business and educational training now more explicitly considers coachability and associated constructs [41].

### Systematic Review Rationale and Aims

Coachability has received widespread recognition in several domains, yet a consensus upon a unified definition remains necessary for comprehensive understanding, as well as providing a foundation for empirical investigation and practical utilisation. Whilst likely understood as a multi-dimensional psychological and behavioural construct, the exact compositional nature of component dimensions also remains to be cohesively identified. The purpose of this systematic review was to (Aim 1) establish a consensus and a common definition of coachability and (Aim 2) identify constituent sub-component characteristics. By analysing the framing, definition and examination of coachability across various domains, this review sought to develop a more comprehensive understanding, identify research gaps and limitations, and thereby establish a foundation for future research and practical applications.

## Methods

The peer-reviewed published literature over five decades (1966–2024) was synthesised using systematic review methods. The review was registered with PROSPERO (Reg No.: CRD42023495817) and used the Preferred Reporting Items for Systematic Reviews and Meta-Analyses [42]. All study screening procedures were performed using Covidence systematic review management software (Covidence, 2020).

### Data Sources

Keywords searches identified articles from the entire contents of six databases: CINAHL, Google Scholar, MEDLINE, PsycINFO, Scopus, SPORTDiscus and Web of Science from 1966 until 31 May 2024. The search process was completed between December 2023 and May 2024. Keyword associated with the construct of interest was: coachab*. the population keywords were: athlete* AND/OR sportsperson* AND/OR student* AND/OR employee* AND/OR entrepreneur* AND/OR coach* AND/OR employer* AND/OR teacher*. the context keywords were: sport* AND/OR recreational sport* AND/OR elite sport* AND/OR education* AND/OR business*. following an initial search, authors (SMG, SC) removed duplicates and screened titles/abstracts. Then full-text studies were independently assessed for final review eligibility (by SMG, SC), with no uncertainty when aligned with inclusion or exclusion criteria.

### Inclusion and Exclusion Criteria

Because of the limited volume of literature, studies were included if they were: (i) empirical (qualitative or quantitative) and were published in a peer-reviewed journal between 1966 and January 2024 (inclusive); (ii) ‘grey literature’ such as dissertations and books between 1966 and January 2024 (inclusive); (iii) explicitly stated examination of coachability; (iv) examined coachability sub-component dimensions within sport, business or educational contexts; and respectively, (v) examined participants with mean ages of 12–64 years (inclusive). All levels of engagement in sport context were included (i.e. recreational to international levels). For business contexts, studies encompassing the examination of the current workforce were included; while in educational contexts, studies conducted at the secondary level and above (i.e. age 12 + years) were considered. Studies conducted outside of these contexts and ages, such as clinical paediatric or post-working career settings, were excluded.

### Data Extraction

Aligned with PROSPERO guidelines and the study’s aims, consistent information was extracted and entered into a summary table (see Table 1). Information extracted included: author(s) names; year of publication; study purpose(s)/aim(s); participant characteristics (if applicable), including age, sex and nationality; study characteristics (i.e. research design, number of participants, data collection methods); study context examined (sport, education, business) and key summary findings (see Table 1). For *Aims 1 and 2*, where a definition of coachability and/or coachability sub-components were discussed or examined within included studies, such information was also synthesised (see Table 2). Identified studies were reviewed with information extraction completed by authors SMG and SC. A third author (PN) acted as an independent, if there was any discrepancy on information extracted.

**Table 1:** Summary of study characteristics from a systematic review of identified studies

Study	Study purpose(s)/aim(s)	Data collection methods	Research design	Participant characteristics	Context examined	Summary of main findings
Ogilvie and Tutko (1966) [1]	Book chapter focused on ‘athletes who resist coaching’	N/A	N/A	N/A	Sport, all age levels of athletes in all sports (USA)	Found coachability problems may result from athlete uniqueness and individuality. Three personalities emerged of non-neurotic athletes who present coaching problems (i) lack of trust for authority, (ii) need for freedom and (iii) 'self-direction
Ogilvie and Tutko (1968) [43]	Examined the effects of high-level competition on personality traits	N/A	The research design included sociometric and psychometric studies	The paper discussed the personality traits of high-level competitors in sports, particularly focusing on age-group swimmers between the ages of 10 and 14 years	Sport, swimming (USA)	Traits such as emotional stability, self-control, and trustfulness, among others, were significantly related to athletic success
Tutko and Richards (1971) [4]	Book chapter focused on personality traits associated with high athletic achievement, particularly emotional factors related to coachability	N/A	N/A	N/A	Sport, all age levels of athletes in all sports (USA)	Coachability was shaped by athletes' emotions, thoughts about themselves, and their relationship with the coaching staff. Athletes were encouraged to ask questions, while coaches provided support and built personal connections with them
Smith et al. (1995) [9]	Evaluated the relative importance of psychological and physical skill. Examined factors predicting performance and survival in professional baseball	Self-reported psychological skills using the Athletic Coping Skills Inventory (ACSI-28) and parallel instruments to obtain psychological skills ratings from coaches and managers	Quantitative descriptive	104 minor league baseball players	Sport, professional level baseball (USA)	Psychological skills, both self-reported and coach-rated, were independent of physical skills and significantly predicted performance and survival in professional baseball over a 2–3-year period. It highlighted the correlations and predictive value of the coachability sub-scale in relation to performance outcomes
Smith et al. (1995) [44]	Aimed to develop and validate a multidimensional measure of sport-specific psychological skills: the Athletic Coping Skills Inventory-28 (ACSI-28)	Conducted principal component analysis of an 87-item instrument, followed by a confirmatory factor analysis of a refined 28-item version (ACSI-28)	Designed as an instrument development and validation study through an exploratory and confirmatory factor analysis	Involved 637 male and female athletes from high school and college teams across multiple sports	Sport, high school and college level (USA)	Confirmatory factor analysis demonstrated the factorial validity of the ACSI-28, which contained seven subscales. Psychometric analyses provided evidence for the construct and predictive validity of psychological skills related to sport performance
Piedmont et al. (1999) [10]	Investigated whether the dimensions of the Five-Factor Model of personality could serve as a predictor of athletic performance	Coaches and individuals completed separate ratings at the end of the season. The data were collected through two primary methods: completion of a bipolar adjective scale to measure the five major dimensions of personality, and coaches’ ratings on several performance dimensions, alongside the collection of actual game statistics	Correlational study with regression analyses	79 female athletes from four different women’s NCAA Division 1 soccer teams	Sport, college level soccer (USA)	Found that the personality dimensions of Neuroticism and Conscientiousness was significantly related to athletic performance among women college soccer players. Neuroticism had a negative correlation with coachability, while conscientiousness had a positive correlation. This suggests that athletes who are less neurotic and more conscientious tend to be more coachable
Giacobbi et al. (2000)^a^ [11]	Developed a measure of athletic coachability and assessed its internal factor structure and reliability	Two semi-structured interviews and author-developed Athletic Coachability Scale	Mixed methods	*N* = 170 Male (*n* = 55) and female (*n* = 115)	Sport, college level basketball, football, golf, swimming, diving, track and field, and soccer (USA)	Identified coachability as a multidimensional construct defined by intensity of effort, trust/respect for the coach, openness to learning, reactions to coaching feedback, working with teammates, and coping with criticism were considered pivotal in predicting athletes' success
Giacobbi et al. (2002) [15]	Investigated college coach perceptions about observed individual athlete traits and personality characteristics who made substantial progress with skill development during their college athletic careers. In addition, team and coach influences on specific athletes was assessed	Interview (semi-structured)	Qualitative descriptive	*N* = 10 NCAA Division 1 Head Coaches across 7 sports	Sport, college level basketball, football, golf, Swimming, diving, track and field, and soccer (USA)	Coaches identified athletes who made substantial progress as being highly competitive/motivated and receptive to instruction. Coachability and developmental characteristics were also deemed as important for the athlete’s progression
Bacon and Spear (2003) [16]	Book purpose was to provide a client-centred approach to performance improvement through adaptive coaching techniques	N/A	N/A	N/A	Business, coaching (North America and Europe)	Clients who actively sought coaching and feedback were deemed more coachable, as they demonstrated a willingness to learn and adapt. Conversely, those who did not seek or resisted coaching tended to hinder their own progress and the potential for positive change
Bebetsos and Antoniou (2003) [46]	Examined age and sex differences in psychological skills among Greek badminton players	Greek version of the Athletic Coping Skills Inventory-28 questionnaire	Quantitative descriptive	*N* = 85 badminton players Male (*n* = 44) and female (*n* = 41)	Sport, national level badminton (Greece)	Showed older players were better prepared to cope with adversity and similar destructive aspects. The older individuals showed a more positive attitude toward complying with the coach’s decision(s) and advice. Study showed no differences by sex. Concluded that the inventory might be a better measure of coping skills, rather than of psychological performance skills
Bourgeois et al. (2003) [47]	Examined relationships between the Balanced Inventory of Desirable Responding (BIDR) indices of impression management (IM) and self-deception (SDE) response styles with the Athletic Coping Skills Inventory (ACSI-28) subscales	Booklet consisting of the BIDR and the ACSI-28	Cross-sectional	*N* = 470 Male (*n* = 210) and female (*n* = 258)	Education, undergraduate psychology and kinesiology (USA)	Suggested that the Athletic Coping Skills Inventory (ACSI-28) subscales was significantly influenced by self-deceptive responding, with coachability being one of the subscales affected
Becker and Solomon (2005) [48]	Examined the impact of coach expectations on athlete performance	Coaches and athletes completed the Coach Effectiveness Questionnaire (CEQ) and Solomon Expectancy Sources Scale (SESS)	Quantitative descriptive	70 head coaches Male (*n* = 34) and female (*n* = 36) 186 athletes Male (*n* = 60) and female (*n* = 126)	Sport, college level basketball (USA)	Psychological factors were the primary sources of information coaches used to evaluate athlete ability. The top five factors were Hard Worker, Receptivity to Coaching, Willingness to Learn, Love of Sport, and Willingness to Listen. Physical sources of information, such as athleticism and coordination, were not in the top one third of items reported. Successful coaches effectively conveyed their expectations to athletes, contributing to team performance
Shannahan (2006)^a^ [49]	Investigated the impact of salesperson coachability, trait competitiveness and leadership style on salesperson performance from an interactionist perspective	A coachability scale was adapted from the sport psychology literature and applied to salespeople in a sales setting	Cross-sectional	*N* = 270	Business, healthcare pharmaceutical (USA)	Salesperson coachability had significantly moderated the relationship between both transformational and transactional leadership styles and salesperson performance. This suggested that coachability is a crucial variable in enhancing sales performance regardless of the leadership style employed
Solomon (2008) [14]	Created a quantitative instrument to identify criteria coaches use to assess athletic ability	Conceptualised Solomon Expectancy Sources Scale (SESS) Three phases of data collection	Quantitative descriptive instrument	*N* = 274 NCAA Division I intercollegiate coaches	Sport, college level 18 sports (USA)	Created a quantitative instrument to identify criteria coaches use to assess athletic ability. Identified 30 qualities in four factors: Coachability, Team Player, Physical Ability, and Maturity
Mitteness et al. (2010) [21]	Developed a model of how entrepreneurs exhibiting key leadership behaviours; trustworthiness, coachability and passion more successfully gain the support of critical stakeholders	Evaluated entrepreneur pitch using a 5-point Likert scale	Quantitative descriptive research design with an observational component	78 different angels who evaluated 205 entrepreneurs presenting their venture ideas between August 2006 and November 2009	Business, Angel Organisation (USA)	Entrepreneurs perceived as coachable are more likely to be viewed favourably by angel investors. Authentic transformational leadership behaviours, such as seeking feedback and being open about weaknesses, enhance perceived coachability and, consequently, improved evaluations of funding potential
Favor (2011) [12]	Examined the relationship between personality traits and coachability in sport teams	Author developed 18-item instrument using a 5-point scale	Mixed-methods cross-sectional survey design	*N* = 36 (head coaches) and *N* = 190 (female college softball athletes)	Sport, college level softball (USA)	Found significant behavioural differences between less coachable (lower-level Cooperation, higher levels of Anger and Immoderation) and more coachable athletes
Navarre (2012)^a^ [50]	Identified the experiences and perceptions of inter-collegiate soccer coaches who coach both male and female teams	Interview (semi-structured)	Qualitative	*N* = 15 male NCAA Division III coaches	Sport, college level soccer (USA)	Twelve higher order themes emerged from the data. The study revealed differences in coach–athlete relationships between male and female athletes, indicating that female athletes were perceived as more coachable, receptive to instruction, and willing to change or try new ideas compared with male athletes
Driska et al. (2012) [8]	Study had two main purposes: (i) determined specific attributes of mental toughness in swimming and (ii) identified situations and environmental factors that lead to mental toughness development in swimmers	Interview (semi-structured)	Qualitative	*N* = 13 Swim coaches Male (*n* = 9) and female (*n* = 4)	Sport, college level swimming (USA)	Mentally tough swimmers demonstrated a high level of receptivity to the coach’s feedback (positive and negative), forming a partnership with the coach. Coachability was linked to bidirectional communication, the development of trust with coaches and a strong buy-in to the team philosophy. The athletes had ‘competitive maturity’ developed through experiences of failure and adversity
Mills et al. (2012) [51]	Identified factors perceived to influence development of elite youth football players	Interview (semi-structured)	Qualitative descriptive	*N* = 10 Expert development coaches	Sport, elite youth football (UK)	Study revealed 23 lower order themes and six higher order categories that collectively represented the factors perceived by expert coaches to influence player development. Coachability (lower order theme) came under Sports Specific Attributes (higher order). Coachable players were able to absorb information presented to them and were receptive to their coaches’ methods
Kirby et al. (2013) [24]	Introduced the concept of salesperson coachability and proposed potential relationships between it and sales coaching and sales performance	Author developed assessment of the adapted Athletic Coachability Scale	Qualitative	*N* = 94	Business, sales (USA)	Found that adapting and applying the concept of athlete coachability to salespeople in a personal selling context provided sales management practitioners and academics a better understanding of how certain salesperson personality traits combined and interacted with certain situational influences to impact sales performance
Shannahan et al. (2013) [13]	Examined the relationship between salesperson coachability, trait competitiveness and sales performance	Data was collected through self-administered paper and pencil questionnaires	Two-part empirical study	*N* = 94 Sales representatives Male (*n* = 65) and female (*n* = 29)	Business, sales in food and beverage (USA)	Found sales performance is highest when salespeople are highly coachable, highly competitive and under transformational leadership
Kruger et al. (2013) [52]	Determined whether the sport psychological profiles of talented 13-year-old sport participants differ from less talented participants	Athlete Coping Skills Inventory for Sport (ACSI-28)	Cross-sectional	*N* = 162 Grade 8 learners with a mean age of 13.2 ± 0.33 years Male (*n* = 105) and female (*n* = 99)	Sport, talent identification general sports (South Africa)	Revealed talented 13-year-old adolescents demonstrated higher means in all seven sport psychological characteristics: coping with adversity, peaking under pressure, goal setting, confidence, coachability and the average coping profile, concentration and freedom from worry. The results indicated that talented adolescents exhibited specific sport psychological characteristics compared with less talented adolescents. Note: only 9 boys and 7 girls were classed as talented compared with 91 boys and 98 girls classed as less talented
Beswick (2015) [3]	Book, Chapter 6: Fostering Coachability. Discussed coachability and how it can be developed by coaching practices	N/A	N/A	N/A	Sport, professional soccer, basketball, NFL (UK; USA; Germany)	Emphasised the importance of a coaching style that balances challenge and support to enhance player coachability. It highlighted that both coaches and players play crucial roles in fostering learning and progress or potentially acting as barriers
Solomon (2015) [53]	Explored the influence of coach optimism on criteria used to evaluate athlete ability. The secondary purpose was to explore expectancy source patterns by sport type and sex	Demographic Questionnaire, Solomon Expectancy Sources Scale and Positive and Negative Expectancy Questionnaire	Quantitative instruments	*N* = 93 NCAA Division I head coaches	Sport, college level basketball, golf (USA)	Coaches higher in optimism prioritised coachability (i.e. willingness to listen, receptivity to coaching, handling pressure, respect). These were vital in the development of athletic ability. Team sport coaches prioritised physical ability in evaluating athletic proficiency
Salomaa (2015) [54]	Examined the following research questions: • How and why is executive coaching used in Global Talent Management (GTM)? • What are the experiences of HR professionals in implementing coaching in GTM programmes?	Interview (semi-structured) conducted in Finnish or in English, face-to-face or virtual	Exploratory: a qualitative multiple-case study approach	*N* = 8 consisted of several nationalities located in five countries on three continents	Business, human resource professionals (Finland; USA; Netherlands; Singapore; Denmark)	Three main factors were found to impact coaching success: adaptability of the coaching process to the coachee’s needs; coachability of the coachee; and the international experience of the coach. Coachability is describes how open the coachee is to being coached. Expatriates’ openness, internal motivation, ability to trust and commitment were identified as important factors influencing coaching success
Larkin and O’Connor (2017) [55]	Tried to understand the attributes perceived as important for identifying skilled youth performance in soccer in Australia	Modified Delphi method with initial interview and two rounds of questionnaires	Modified Delphi method	*N* = 20 coaches	Sport, talent identification soccer (Australia)	Recruiters perceived technical, tactical and psychological attributes as important for youth soccer performance. The main findings highlighted the perceived importance of coachability and a positive attitude as crucial psychological attributes for identifying skilled youth performance in soccer
Ciuchta et al. (2018) [19]	Established and validated the entrepreneurial coachability construct and measurement scale	Author developed and validated a measurement scale for entrepreneurial coachability	Qualitative descriptive	*N* = 93 NCAA Division I head coaches	Business, entrepreneurship practice (USA)	The study found that entrepreneurial coachability serves as a viable signal influencing potential investors and has predictive power on funding outcomes, demonstrating its importance in entrepreneurial pitches and investment decisions
Mayer et al. (2018) [56]	Examined the relationship between emotional intelligence, coachability and organisational citizenship behaviour to determine if emotional intelligence and coachability can increase organisational citizenship behaviour	Survey-based methods: Section I consisted of demographic information, Section II consisted of the Athletic Coachability Scale; Section III consisted of the Assessing Emotions Scale; Section IV consisted of the Organizational Citizenship Behaviour Scale	Quantitative	*N* = 106 MBA students	Business, organisational citizenship behaviour (USA)	Individuals with higher levels of coachability were more likely to exhibit organisational citizenship behaviour, showcasing the importance of coachability in fostering positive work outcomes and organisational success. The results indicated positive relationships, which suggest it may be desirable for higher education institutions to help students develop these soft skills, and organisations should consider incorporating soft skills assessment as part of their hiring strategy
Weiss (2019)^a^ [57]	Examined the importance and impact of coachability in coaching interactions. Tried to determine the relationship between employee coachability and job performance, adaptability and promotability	Survey-based methods Each coachee completed one survey, whereas coaches completed one survey for each of their coachees	Cross-sectional	*N* = 327	Business, pharmaceutical organiaation (USA)	Coachability behaviours were important for optimising coaching interactions. Employee coachability drove individual job performance, adaptability and promotability. Employee adaptability was a vital driver of organisational effectiveness. Organisations may consider employee coachability as a competency to achieve competitive advantages
Marvel et al. (2020) [20]	Investigated how founder human capital and founder coachability relate to exploiting product innovation in new ventures	Data from 59 different countries collected through separate confidential surveys completed by startup coaches and founders	Entrepreneurial qualitative case study	*N* = 401 startup coaches and *N* = 686 founders completed individual surveys	Business, emerging organisations (USA)	Coachability significantly boosted product innovation, as highlighted by scholars and practitioners. It served not only as a signal to stakeholders but also as a valuable learning mechanism for entrepreneurs to acquire and integrate new knowledge effectively
Kuratko et al. (2021) [58]	Examined mentors and founders across entrepreneurial support organisations to investigate the factors that influenced an entrepreneur’s coachability, how it translated to venture outcomes and if the mentor–mentee relationship met entrepreneur expectations	Data were collected from startup mentors and founders in two survey waves: Sept 2018-Jul 2019 and Nov-Dec 2019, focusing on backgrounds, coachability, venture development and relationships	Empirical research study	*N* = 401 startup coaches and *N* = 686 founders completed individual surveys	Business, entrepreneurs (USA)	Founders with higher entrepreneurial self-efficacy were more satisfied with mentorship. Female founders were seen as more coachable. Coachability positively impacted venture progress and success. Additionally, coachable entrepreneurs had better relationships with mentors, with learning goal orientation enhancing mentorship satisfaction
Wu et al. (2021) [59]	Examined the relationship between dispositional mindfulness, psychological skills and mental toughness in multiple-sport populations	Self-reported questionnaires: Chinese Version Mindful Attention Awareness Scale (CMAAS), Athletic Psychological Skills Inventory (APSI), Trait Mental Toughness Inventory for Sport (TMTIS)	Cross-sectional	*N* = 101 College athletes Male (*n* = 72) and female (*n* = 29)	Sport, college level Taekwondo, Judo, archery, Wushu, tennis (Taiwan)	Found a positive correlation between dispositional mindfulness and psychological skills, as well as mental toughness among college athletes. The findings suggested that higher dispositional mindfulness may contribute to improved coachability, potentially enhancing athletes’ performance and coping abilities under pressure
Maxheimer and Nicholls-Nixon (2021) [60]	Explored the dimensions of coaching relationships that impact women entrepreneurs during business incubation, providing deeper insight into their coaching needs	Interview (semi-structured)	Qualitative	*N* = 15 Male (*n* = 5) Female (*n* = 6) Coaches (*n* = 4)	Business, entrepreneurship (Canada)	Female entrepreneurs placed different emphasis on coaching dimensions, such as venture support, emotional support and sex inclusivity, compared with men, influencing their entrepreneurial self-efficacy and outcomes during business incubation. It highlighted the significance of coachability, indicating that the willingness and openness of entrepreneurs to receive support and integrate feedback from coaches play a vital role in impacting their entrepreneurial outcome
Nguyen et al. (2021) [61]	Explored coaches’ beliefs about the role of child and adolescent shyness in team sports	Coaches completed two open-ended questions assessing their beliefs about the benefits of team sports participation for shy children and adolescents, as well as the special contributions that shy team members may make to a sports team	Entrepreneurial qualitative case study	(*N* = 496) coaches Male (*n* = 403) and female (*n* = 93)	Sport, amateur level team sports; hockey, soccer, football, basketball, baseball, Ringette, Lacrosse, rugby (Canada)	Coaches perceived shy athletes as more coachable, observant and attentive, based on their specific characteristics and contributions to the team
Davis (2021)^a^ [62]	Examined coachability from the perspectives of athletes and coaches, created a model of coachability from both perspectives	Interview (semi-structured)	Exploratory qualitative case-study design	*N* = 12 (*n* = 4 NCAA men’s head coaches, *n* = 8 male athletes)	Sport, college level basketball (USA)	A model of coachability was created based on the participants’ responses gathered in the interviews. The model created could be useful to coaches for identifying and recruiting athletes with traits, attributes and other key factors that will enhance their coachability (coach awareness, trusting relationship, willingness to learn, mutual respect)
Johnson et al. (2021) [17]	Developed a conceptualisation and measure of workplace coachability	Four samples consisting of online questionnaires: self-reported and supervisor ratings	Quantitative	Sample 1: *N* = 99 employees and *N* = 89 pairs Sample 2: *N* = 306 Sample 3: *N* = 209 Sample 4: *N* = 179	Business, numerous industries (USA)	Unveiled a comprehensive conceptualisation of coachability and demonstrated its significance and implications for workplace coaching. The Coachability Scale could provide insight into the likelihood that an individual will be receptive to coaching and on which dimension(s) the individual might need additional support. Therefore, the measure has the potential as a diagnostic tool for identifying the coachee’s needs and which coaching methods would best suit him or her
Weiss and Merrigan (2021) [23]	Explored whether employee coachability drives employee job performance, adaptability and promotability	Two questionnaires were created: one for direct reports (coachees) and one for managers (coaches)	Cross-sectional	*N* = 450	Business, pharmaceutical organisation (USA)	Demonstrated highly coachable employees achieve greater individual job performance, are significantly more adaptable and are viewed as more promotable. Advised organisations should identify highly coachable individuals during the hiring process and elevate the skill sets of new hires and existing employees to become even more coachable
Kraska (2021)^a^ [63]	Explored the relationships between personality traits and athletic performance in college athletes	Personality was measured through the 16 Personality Factor Questionnaire (6th Edition). Athletic performance was measured through 1. coach evaluations (Coach Rating Scale) and 2. a calculated performance statistic (percent of games started in the season)	Cross-sectional	*N* = 85	Sport, college level soccer and volleyball (USA)	Found athletic ability was the strongest predictor of athletic performance. It identified athletic performance was a multifaceted construct consisting of coachability, athletic ability, game performance, team player, work ethic and the composite score of these domains
Iwasaki et al. (2022) [45]	Examined the mediating role of mindful engagement in the relationship between male high school athletes’ motivational climate perceptions on their teams (i.e. caring, task, and ego involving climate) to athlete coachability	Questionnaires: Caring Climate Scale, Perceived Motivational Climate in Sport Questionnaire, Short version of CAMS-R: Mindful engagement, the Athletic Coping Skills Inventory-28	Quantitative descriptive	*N* = 164 All male	Sport, high school level soccer, football, basketball and cross-country (USA)	Perceptions of a caring and task-involving climate were significant antecedent variables for mindful engagement and coachability, while perceptions of an ego-involving climate showed a negative relationship with these factors. Mindful engagement can foster a supportive and encouraging environment and may enhance athletes’ ability to be coachable
Somià (2022) [64]	Developed and assessed the coachability competency model for entrepreneurship education, aiming to provide a tailored approach for supporting students transitioning from student to entrepreneur	The Coachability Competency Survey and the Behavioural Event Interview (BEI)	A multi-perspective approach Mixed-methods, quantitative and qualitative	*N* = 450 final number of participants	Education, entrepreneurship education (EU funded, study location not specified)	This multi-dimensional perspective enhanced the understanding of coachability and its application in diverse contexts, highlighting its relevance in the development of entrepreneurial competencies from student to entrepreneur adopting a competency-based approach in assessing and training coachability
Bar-On and VanDijk (2022) [65]	Created a comprehensive model and measure of performance by evaluating 18 Core Factors and 5 Ring Factors that contribute to performance	Online psychometric instrument, the MMP (The Bar-On Multifactor Measure of Performance)	Quantitative descriptive	*N* = 3039	Business, occupational performance in small-, medium- and large-sized organisations (global English-speaking countries)	The Multifactor Measure of Human Performance effectively identified and offered developmental suggestions for individual’s performance, including their readiness to enhance performance through self-development efforts. The assessment included a scale for coachability, measuring an individual’s readiness to commit to enhancing their performance through developmental efforts
Azim et al. (2023) [66]	Examined the psychological skills of State University of Malang athletes who took part in POMPROV 2022 using ACSI-28	ACSI-28 questionnaire	Quantitative descriptive	*N* = 45	Sport, university athletes across 10 sports (Indonesia)	Psychological factors played a crucial role in athletes’ success. Regarding coachability, athletes were urged to embrace criticism as an opportunity for learning. The study advocated for educating athletes on humility and self-reliance
Weiss et al. (2023) [67]	Identified antecedents that influence coachability represented as a second order factor	Multiple questionnaires	Cross-sectional, survey-based design	*N* = 450 final number of participants	Business, employee coachability (USA)	Identified key underlying individual differences along with multiple environmental factors influencing individuals’ coachability. These proved vital for achieving critical organisational outcomes. The authors encouraged organisations to develop current employees to highly coachable levels through targeted skills training
Tetteh et al. (2023) [68]	Aimed to understand the levels (i.e. mild vs intense) of task conflict expressions between angel investors and entrepreneurs at the post-investment stage and how it affects angel investors’ follow-on investment intentions with the same entrepreneur	Survey	Conjoint experiment	*N* = 71 Male (*n* = 65) and female (*n* = 6)	Business, angel investors and entrepreneur (China)	Angel investors appraisal of task conflict events depended on the degree to which conflicts (mild or intense) were expressed and their perception of the entrepreneurs’ coachability. The perception of entrepreneurs’ coachability significantly influenced angels’ appraisal of task conflict events and subsequently impacted their reinvestment intentions
James-Boone (2023)^a^ [69]	Analysed the notion of professional development coachability from the perspective of experienced professional coaches; define it specifically in the context of professional coaching, and provide a framework for attributes/traits that may be used to establish a coachability measure	Mixed method survey and semi-structured interviews	Exploratory study—grounded theory design used for investigation and analysis	Study 1 (survey, *n* = 209) Study 2 (semi-structured interviews/mini survey, *n* = 44)	Business, professional development coaching in organisational setting (North America 88%; UK 11%)	Coachability in professional development was a dynamic state influenced by internal attributes (curiosity, feedback receptivity, self-awareness, self-determination, self-improvement) and supportive external environments. It changed based on the coachee’’s circumstances and the coaching relationship. Organisations needed to foster environments that nurtured these attributes to support coachability
Jooste et al. (2023) [70]	Investigated the relationship between emotional intelligence and coping ability in female field-hockey players. Determined if emotional intelligence can be a predictor of coping ability. Explored the potential use of emotional intelligence testing and intervention in sport psychology practice	Correlational research design. Convenient sampling method. Pen-and-paper survey used for data collection Emotional Intelligence Scale and Athletic Coping Skills Inventory-28	Cross-sectional	*N* = 60 female National/elite (*n* = 26) University/sub-elite (*n* = 34)	Sport, national/elite and university/sub-elite field hockey (Africa)	Developing emotional intelligence complemented conventional mental skills training for field-hockey players, providing proactive behaviour that helped them better cope with adversity, failure and stress in their sport
Svetek (2023) [71]	Assessed the dimensions used by investors to evaluate entrepreneurs seeking early-stage financing	Conjoint experiment using interview and questionnaire	Conjoint experiment	*N* = 84 (*n* = 44 angel investors) and (*n* = 40 venture capitalists)	Business, entrepreneurship early-stage financing (24 European countries)	Early-stage investors prioritised competence and cooperativeness, with coachability being particularly valued. Entrepreneurs who signalled both traits, especially coachability, enhanced their perceived funding potential. Additionally, good market knowledge could offset limited entrepreneurial experience, and involved investors slightly preferred coachable entrepreneurs
Lewtin et al. (2023) [72]	Explored the role of entrepreneurial coachability in attracting mentors and investors	A review paper	Narrative review	N/A	Business, entrepreneurship (not relevant)	The role of entrepreneurial coachability in venture outcomes gained attention, evidence suggested that investors favoured more coachable entrepreneurs. It remained uncertain whether enhanced coachability directly correlated with improved venture success. Future research should explore this link, while entrepreneurs and coaches could benefit from adopting a coachability perspective to enhance their relationship and potential outcomes
Kemarat et al. (2023) [73]	Investigated the influence of mental skills on competitive anxiety in professional futsal players. To measure the differences in mental skills between successful and unsuccessful players	Questionnaires: the Sport Competitive Anxiety Test (SCAT) and the Athletic Coping Skills Inventory (ACSI-28). Data collection was carried out at the training site 1 day before the match, which was the last competition of each team	Quantitative descriptive	*N* = 80 Participants involved 10 players each from four top (deemed successful) and four bottom ranking teams (deemed unsuccessful)	Sport, professional futsal (Thailand)	Competitive anxiety in the unsuccessful group was affected by freedom from worry and coachability. In contrast, successful players were more open-minded, learned from instruction and feedback (coachability), which helped increase self-confidence and thereby reduce anxiety during competition. Successful players also had higher scores in goal setting and mental preparation
Champatiray et al. (2023) [74]	Explored the existence of a relationship between emotional intelligence and decision making with the mediating role of coachability	Survey Coachability Quotient questionnaire and the Schutte Self-Report Emotional Intelligence Test (SSEIT)	Quantitative descriptive	*N* = 102	Business, financial investors (India)	Examining group dynamics revealed the significance of coachability and emotional intelligence in various contexts. The effects of behavioural treatments on emotional intelligence and coachability warranted empirical investigation. Additionally, comparative studies evaluated the impacts of different coaching methodologies on investor decision making
Fournet (2023)^a^ [75]	Established a theory of individual coachability by (i) defined individual coachability and (ii) empirically evaluated its elements	The author developed a 25-item individual coachability scale	Three-part experimental vignette design Quantitative descriptive	*N* = 254	Business, emerging professionals, upper-level business students (USA)	Author developed a theory of individual coachability, conducted experiments on coaching programme quality, and observed its impact on job performance, employee engagement and organisational commitment
Somia et al. (2024) [41]	Assessed and developed coachability in entrepreneurship education through the use of quantitative and qualitative methods to better understand its impact on student performance and learning	A coachability survey and BEI	Mixed-method design	*N* = 30 students coachability survey *N* = 13 interviewed using the BEI methodology	Business, entrepreneurship education and startup ecosystems (USA)	Emphasised the critical role of both self-awareness and implementation competencies in coachability development and effectiveness. Entrepreneurship education programmes should focus on improving coachability competencies by cultivating self-reflection and self-assessment among students, challenging them to take the initiative, creating and crafting something new, while remaining flexible and adaptable to user feedback and changing circumstances
Ober et al. (2024) [76]	Developed a coachability situational judgement task that appears to be the first attempt to assess coachability through non-Likert-type items	Author developed a 22-item coachability situational judgement task designed to measure participants’ coachability across five workplace-based scenarios. Participants also completed the Workplace Coachability Survey (WCS) along with high and low judgement of job performance	Quantitative instrument	*N* = 800 (age 18–55 years)	Business and education, job performance (USA)	Developed coachability situational judgement task, first non-Likert coachability assessment. Longitudinal research is imperative to assess the potential evolution of coachability over time, whether through natural development or intentional intervention. This could help identify which components of coachability are more malleable than others, discern the mechanisms driving change (mediators) and identify the conditions that amplify these changes (moderators)

*ACSI-28* Athletic Coping Skills Inventory-28, *BEI* Behavioural Event Interview, *BIDR* Balanced Inventory of Desirable Responding, *CEQ* Coach Effectiveness Questionnaire, *Div* Division, *EU* European Union, *IM* impression management, *N/A* not applicable, *NCAA* National Collegiate Athletic Association, *SDE* self-deception enhancement, *SESS* Solomon Expectancy Sources Scale

^a^Grey literature (e.g. dissertations)

**Table 2:** Identified definitions of coachability and constituent components

Study	Coachability definition	Constituent components discussed/identified
Ogilvie and Tutko (1966) [1]	First time ‘coachability’ as a term was described but without explicit	‘Openness to learn’ and ‘receiving coaching instruction’
Ogilvie and Tutko (1968) [43]		‘Open to authorities and have a basic respect for the instruction of others’
Tutko and Richards (1971) [4]		Respects the coach and accepts their advice
Smith et al. (1995) [9]	“… Open to and leams from instruction; accepts constructive criticism without taking it personally and becoming upset” (p. 402)	‘Learning from instruction’ for technical and psychological skill development
Smith et al. (1995) [44]		‘Coping with a setback’, ‘listening to instruction’
Piedmont et al. (1999) [10]	“… Players ability to listen\ learn and apply coaches instructions”. (p. 772)	‘Listening’ and ‘implementing feedback’ from coach
Giacobbi et al. (2000)^a^ [11]	“… as a multidimensional, sport-specific construct characterised by motivation to improve one’s sport skills, inquisitiveness, openness to learning, and trust in and respect for the coach and his or her training process.”	Described coachability as a ‘sport-specific’ construct
Giacobbi et al. (2002) [15]	“… being “receptive to instruction,” “willing to make changes,” “organised,” “more educable,” and “open.” (p. 170)	‘Highly motivated’ and ‘receptive to coach instruction’
Bacon and Spear (2003) [16]		‘Client’s ego strength’, ‘seeks and open to feedback’, ‘awareness of a need to change’, ‘trust in coach’
Bebetsos and Antoniou (2003) [46]	“I listen carefully to the advice and instructions of my coach.” (p. 1291)	‘Willingness’ of an athlete to ‘listen’ carefully to the advice and ‘instructions of their coach’
Bourgeois et al. (2003) [47]	“… Open to and learns from instruction; accepts constructive criticism without taking it personally and becoming upset” (p. 74)	‘Receptive to coach instruction’
Becker and Solomon (2005)[48]		‘Handling pressure’ and ‘respect’
Shannahan 2006^a^ [49]	“… as the ability to accept and implement feedback.” (p. 27)	Described as a situation-specific construct
Solomon (2008) [14]		‘Receptivity to coaching’, ‘willingness to listen’ and ‘willingness to learn’
Mitteness et al. (2010) [21]	“… the degree to which entrepreneurs listen to key stakeholders, carefully consider feedback prior to responding, recognize their weaknesses, and willingly make changes to address those weaknesses.” (p. 4)	Referred to as ‘balanced processing of information’ and ‘seeking out and listening to feedback on their weaknesses and vulnerabilities’
Favor (2011) [12]		‘Willingness to listen’, learn, change; emotional maturity; determination and commitment; trust and respect for coaches; reaction to feedback and instruction; positive interactions with others
Navarre (2012)^a^ [50]	“… as a construct that includes (but is not limited to) an athlete being inquisitive, teachable, attentive and receptive to instruction, trusting, and willing to change or try new ideas, concepts, and strategies.” (p. 164)	‘Listening’, ‘trusting coach’,‘motivated’, ‘feedback implementation’
Driska et al. (2012) [8]	“… to be receptive to the coach’s feedback (positive and negative), to communicate effectively with the coach, and to develop a partnership with the coach.” (p. 194–5)	Characterised as an attitude/ mindset to: (a) athlete buy-in to team philosophy and developing trust with the coaching staff, (b) effective bidirectional communication between coach and athlete, and (c) a strong desire to grow from every athletic experience
Mills et al. (2012) [51]		‘Willingness to listen’, ‘hunger to learn’, ‘consistency’ and ‘take it forward’
Kirby et al. (2013) [24]	“… is willing to provide information to the sales manager/sales coach; exhibits trust and respect towards the sales manager/sales coach; is flexible and adaptable to changes in routing; and seeks feedback and information from other sources to improve his/her selling skills.” (p. 413)	Considered dimensions of salesperson coachability as: ‘intensity of effort’, ‘openness to learning’, ‘reactions to manager/coach’s feedback’, ‘trust/respect for the manager/coach’, ‘working with teammates’, ‘coping with criticism’
Shannahan et al. (2013) [13]	“…. as the degree to which salespeople are open to seeking, receiving, and using external resources to increase their sales performance in a personal selling context.” (p. 41)	Characterised as an interactional variable. It can vary because contextual features (the environment) will influence the manifestation of their coachable components
Kruger et al. (2013) [52]	“…. as the ability to handle constructive criticism and not take it personally, as well as the ability to listen to instructions and learn skills.” (p. 653)	Referred to as a predictor of sports performance
Beswick (2015) [3]		‘Ability or willingness to listen’, ‘keen to learn’, ‘trusts and empowers coach’, ‘willingness to try new things’, ‘adapt to change’, ‘accept accountability’, ‘unafraid of mistakes’, ‘inquisitive’, ‘positive response to instruction or criticism’, ‘safe learning environment’
Solomon (2015) [53]	“Willingness to learn, respect, handling pressure, competitive demeanour” (p. 103)	‘Willingness to listen’, ‘receptivity to coaching’, ‘handling pressure’ and 'respect’
Salomaa (2015) [54]	“… how open the coachee is to being coached.” (p. 228)	‘Openness’, ‘internal motivation’, ‘ability to trust’ and ‘commitment’
Larkin and O’Connor (2017) [55]	“… Ability to be coached; willing to learn; coachable; good learners; responsive to coaches.” (p. 5)	‘Willingness to listen to the coach’, ‘a desire to learn new skills even in the face of adversity (not deterred by a setback or mistake)’
Ciuchta et al. (2018) [19]	“… the degree to which an entrepreneur seeks, carefully considers, and integrates feedback to improve his or her venture’s performance." (p. 861)	‘Openness to learning new skills’, ‘intensity of effort’, ‘trust and respect’, ‘performance driven’
Mayer et al. (2018) [56]	“… as the ability of a person to receive and use constructive criticism and feedback to improve their workplace performance.” (p. 4)	‘The ability to accept and implement feedback’, ‘eager to learn’, ‘listen to new ideas, thoughts, feedback or perspectives’, ‘reflect (at their work, behaviours, and attitudes and question if they are affecting those around them)’
Weiss (2019)^a^ [57]	“… an individual difference influencing the degree to which employees are open to seeking, receiving, and using coaching feedback to drive individual development and improve performance.” (p. 74)	‘Feedback seeking, feedback receptivity and implementation of feedback behaviours’
Marvel et al. (2020) [20]	“… as a learning mechanism beneficial for exploring new knowledge, while also alleviating the constraining effects of prior knowledge, to create innovative offerings.” (p. 12)	‘Seeking out’ and ‘exploring new knowledge’
Kuratko et al. (2021) [58]		‘Proactively seeking feedback’, ‘listening’, ‘willingness to learn and be coached’, ‘receptive to constructive feedback’ and ‘cognitive adaptability’
Wu et al. (2021) [59]	“… refers to the attitude of athletes adapting and obeying their coach’s leadership and guidance and accepting the coach’s constructive criticism.” (p. 5)	Considered an ‘attitude’
Maxheimer and Nicholls-Nixon (2021) [60]	“… refers to the extent to which an entrepreneur is willing and open to receiving support and integrating feedback provided by coaches.” (p. 567)	‘Receptive and open to feedback’
Nguyen et al. (2021) [61]		Listed behaviours of: ‘observant, attentive and rule abiding’
Davis (2021)^a^ [62]	“… as being accepting of criticism and having a behaviour and actions that demonstrate a willingness to learn and accept feedback from coaches” (p. 122, NSCAA, 2013)	‘Supportive family’, ‘accepting constructive criticism’, ‘willing to listen’, ‘willingness to learn’, ‘receptive to coach feedback/instruction’ and ‘implementation’
Johnson et al. (2021) [17]	“… as a tendency to be comfortable working with and willing to learn from a coach.” (p. 585)	‘Comfort with coaching’, ‘development orientation’, ‘acceptance of feedback’, ‘feedback humility’, ‘emotional reaction to feedback’, ‘orientation toward negative feedback’
Weiss and Merrigan (2021) [23]	“… an individual difference influencing the degree to which employees seek, receive (i.e., demonstrate receptivity to), and implement constructive feedback to drive individual development and improve performance.” (p. 124)	‘Feedback seeking’, 'feedback receptivity’, and 'implementation of feedback’
Kraska (2021)^a^ [63]	“… the player's ability to listen, learn and apply coaches instructions.” (p. 45)	‘Listening’ and ‘implementing coach instruction’
Iwasaki et al. (2022) [44]		‘Self-regulate their attention in the moment’, ‘mindful engagement’, ‘listening carefully to the advice and instruction of their coaches’
Somià (2022) [64]	“… as a multi-dimensional construct, which manifests itself in the exhibition of specific behaviours.” (p.8)	‘Learning’ aspect consisting of ‘openness/willingness to learn’; ‘the holding of a developmental orientation’ and, a degree of ‘commitment and ‘intensity of effort towards improving one’s skills and competencies’
Bar-On and VanDijk (2022) [65]	“… suggests how readily and meaningfully individuals are expected to respond to efforts designed to enhance their performance. It implies how well they might benefit from investing in coaching, mentoring or group training for the purpose of further self-development.” (p. 9)	‘Responding to efforts designed to enhance their performance’ and ‘open to being coached for the purpose of further self-development’
Azim et al. (2023) [66]	“… always accept learning in the form of criticism and suggestions, thirst for practice and high curiosity, have an open heart even though criticism and suggestions are done in a painful way.”	‘Humility’ and ‘self-sufficiency’
Weiss et al. (2023) [67]		‘Feedback orientation’, ‘expressed humility’ and the ‘instrumental feedback motive’
Tetteh et al. (2023) [68]		Conceptualised resistance to coachability: ‘as the entrepreneur’s unwillingness to seek, consider and integrate feedback’. Instead ‘listen and process feedback’
James-Boone (2023)^a^ [69]	“… as an amalgamation of external and internal factors that align at a specific moment under specific circumstances. Therefore, it is complex, highly malleable, and impacted by conditions in and around the coaching engagement, both internal and external to the coachee.” (p. 90)	Referred to five internal factors ‘curiosity, feedback receptivity, self-awareness, self-determination/motivation and self-improvement’
Jooste et al. (2023) [70]		‘Receptivity to feedback’, ‘coach instruction essential for sport specific learning in less skilled athletes’
Svetek (2023) [71]		Utilised traits of: ‘competence (persistence, commitment, resilience)’ Attributes of low coachability: the entrepreneur is sometimes receptive to the investors’ feedback but does not seek it. Attributes of high coachability: the entrepreneur actively seeks the investors’ feedback and takes it carefully into account
Lewtin et al. (2023) [72]		Considered coachability as a two-way street, it’s a dynamic relationship between coach/manager and coachee
Kemarat et al. (2023) [73]		‘More open-minded’ and ‘learned from instruction’ and ‘feedback’ which helped increasing ‘self-confidence’ thereby reducing anxiety during playing
Champatiray et al. (2023) [74]		‘Information processing’, ‘decision-making’ and ‘emotional intelligence’
Fournet (2023)^a^ [75]	“… as the degree to which an individual seeks a desirable and sustainable change, integrating goal-setting, self-efficacy, feedback, and accountability.” (p. 28)	‘Goal-setting’, ‘self-efficacy’, ‘feedback-seeking’ and ‘accountability’
Somia et al. (2024) [41]	“… is a bundle of competencies that allow the achievement of coaching goals: starting from self-awareness and commitment, developing learning and relationship management competencies, and implementing what is learned from the coaching relationship.” (p. 3)	‘Self-awareness’, ‘commitment’, ‘learning’, ‘relationships’ and ‘implementation’
Ober et al. (2024) [76]	“… conceptualized as an individual difference construct describing how individuals seek, engage with, process, and react to performance feedback (both positive and negative) and other learning opportunities.” (p. 1)	Coachability is conceptualised as a continuous variable with components of: ‘feedback orientation’, ‘learning orientation’, ‘relationships with coaches’, ‘learning initiative’, ‘knowledge and skill self-awareness’ and ‘growth mindset orientation’
Interviews with diving coaches^b^	“… ability to receive and understand instruction (cognitive) leading to a mechanical (behavioural) change, not deterred by a setback and have a willingness to learn in order to continually improve”	‘Attentiveness’, ‘willingness to continually learn’, ‘comprehend instruction’, ‘recognise movement error’, ‘seeks feedback’, ‘overcome setbacks/failure’, ‘receptivity to feedback’, ‘athlete drive’
Interviews with hockey coaches^b^	“… is curious to discover and eager to learn, receptive to feedback, willingness to implement the feedback and a resilience to keep trying.”	‘Willingness to learn’, ‘seeks feedback’, ‘attentive to technical skills’, ‘attentive to tactical awareness’, ‘persistence’, ‘engagement’, ‘implementing feedback’

^a^Grey literature (e.g. dissertations)

^b^Semi-structured interviews conducted with *N* = 8 coaches (*n* = 5 diving; *n* = 3 field hockey)

### Semi-Structured Interviews

To supplement synthesised information from research studies and validate findings, expert coach practitioner understanding from two sporting contexts were consulted. Authors (SMG, SC) conducted two independent semi-structured focus-group interviews with elite diving (*n* = 5) and field hockey coaches (*n* = 3). Coaches had extensive experience in their respective context domains, having coached for an average of 10 years (standard deviation = 3.0) at state, national and Olympic levels, with both developmental and senior adult (male and female) athletes. Aligned with *Aims 1* and *2*, within interviews coaches were invited to (i) define and describe coachability as relevant to their sport context and athletes, (ii) identify coachability characteristic features, (iii) identify examples of higher and lower coachability, and (iv) identify behavioural features of coachability in training and/or competition contexts.

## Results

### Summary of Identified Studies

**Fig. 1:**
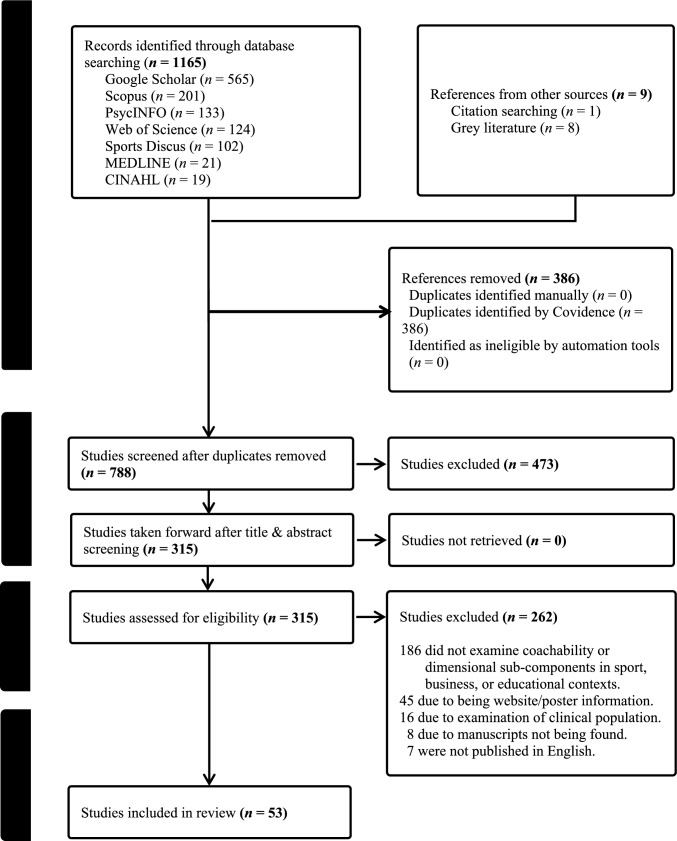
Flow diagram summarising the screening and selection of studies according to preferred reporting items for systematic reviews and meta-analyses [42]

The initial search yielded 1165 potential studies. For a summary of the screening and selection process, see Fig. 1. Initially identified studies were then excluded for (i) being duplicates (*n* = 386), (ii) not meeting title and abstract screening (*n* = 473), (iii) did not examine coachability or dimensional sub-components in a sport, business or educational context (*n* = 186), (iv) were website information or posters (*n* = 45), (v) examined clinical populations (*n* = 16), (vi) the full article could not be tracked/identified (*n* = 8), and (vii) the article was not published in English (*n* = 7), with 1121 studies eventually excluded. Nine references were identified from other sources: one from citation searching (*n* = *1*) and eight from the grey literature (*n* = *8*), of which four of the remaining grey literature studies were identified through the initial search.

Fifty-three articles met the inclusion criteria (see Table 1), including 41 peer-reviewed journal publications, 4 book sub-sections/chapters and 8 PhD dissertations. Studies were associated with four main disciplines: sports psychology and sports coaching (*n* = 27), business occupations (*n* = 13), business and entrepreneurial contexts (e.g. angel investing, entrepreneurship education; *n* = 9), and education (*n* = 4). Of the 53 included articles, 43 reported their country of origin: 70% were conducted in the USA (*n* = 32), with others in Canada (*n* = 2), South Africa (*n* = 2) and one each in Australia, China, Greece, India, Indonesia, Thailand and the UK. The remaining ten studies did not specify a country location of study.

The identified literature across disciplinary contexts of investigation had varied study aims(s) and objective(s), and which evolved over time. For instance, early sporting studies explored associations between personality traits or individual characteristics with high-level competitive success [43]. Whereas from the mid-late 1990s, sporting studies often utilised psychological assessments to understand whether coachability and associated component constructs were associated with sporting performance [10, 15, 45]. Researchers also examined criteria used by coaches to evaluate an athlete’s ability [14, 47], investigating whether, and which, psychological components including coachability were considered. More recently, studies have shifted toward understanding the impact of coachability on performance, athletic development and interpersonal dynamics [10, 45, 53, 55] as well as how coaching conduct and relationships impact coachability [51].

Initial studies in business contexts examined relationships between salesperson coachability and sales performance [13, 24], before expanding to examine the value of occupational coachability more broadly. In parallel, the creation and validation of coachability measurement scales for employee evaluation and professional development has occurred [17, 19, 23]. More recently, research has investigated how entrepreneurial coachability relates to the capability to attract business mentors and secure financial investment [71, 72]. Meanwhile in education contexts, recent efforts have focused on creating models and practical assessments to enhance student learning [76] and entrepreneurial transition [41, 64].

### Aim 1: Coachability Defined

Across and within identified studies, coachability was variably defined but with a common view of being considered as a multi-component construct (see Table 2). Definitions ranged from coachability reflecting an underlying stable cognitive attitude (e.g. [58]) to being more frequently considered as a malleable mindset or meta-cognitive frame (i.e. openness, receptive [11, 19, 23]). Relative to the earlier literature, contemporary definitions considered coachability as an inter-individual difference [76], where both cognitive and behavioural features were learned, developed and intentionally implemented [70]. Coachability was described as encapsulating a “*bundle of competencies*” [41], which could be holistically or individually considered on a low–high continuum, and where degrees [21] or levels of capability can be observably demonstrated [56].

Most commonly, definitions focused on behaviours, referring to observable engagement, responses or actions in specific learning and/or performance situations. Studies described coachability as comprising an inherent motivation and a ‘willingness to learn’, being inquisitive, curious and committed to personal improvement [62, 64, 71]. Coachable individuals were characterised as those who listened (*n* = 5 definitions), were open and positively receptive to (critical/constructive) feedback (*n* = 8), actively sought feedback (*n* = 6) and actively implemented/actioned feedback (*n* = 4) to either address skills/task weaknesses or enhance learning and development. Further, despite encountering setbacks, coachable individuals were described as exhibiting perseverance, remaining focused on skill/task improvement without deterrence from learning setbacks or challenges (e.g. [17], coach interviews).

### Aim 2: Coachability Constituent Components

**Fig. 2:**
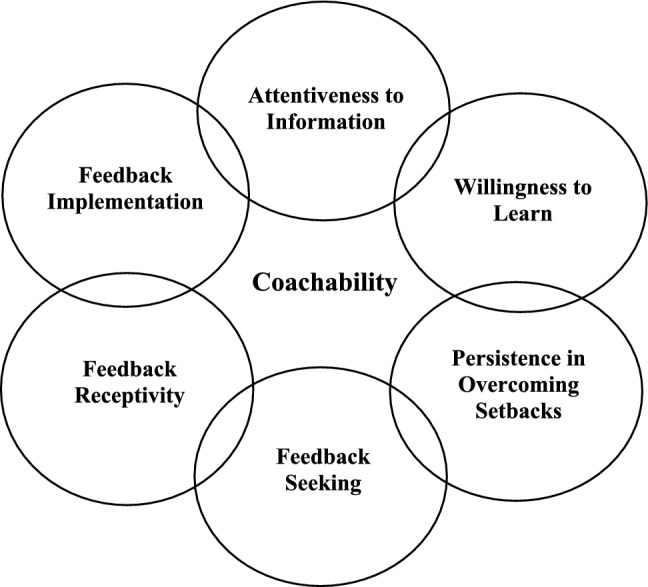
Visual summary of coachability constituent components according to the identified literature in sport, business and education domains

Through a review of identified studies and evaluation of definitions utilised, six consistent constituent coachability components were identified (see Table 2). Listed in alignment with the interactional processes of receiving, decision making, and subsequently enacting or responding to information, these were termed: *Attentiveness to information, Willingness to learn, Persistence in overcoming setbacks, Feedback seeking, Feedback receptivity and Feedback implementation* (see Fig. 2).

#### Attentiveness to Information

Considered a critical initial process, attentiveness to information was consistently identified in 22 articles (42%) as a constituent component [3, 9–12, 14–16, 45, 46, 50–53, 55, 56, 58, 61–63, 68, 76]. Across investigation domains, attentiveness was predominantly associated with forms of listening to the advice and instructions from coaches/mentors [9, 15, 46], and to a lesser extent attention toward external adjunctive information sources [3]. Active intentional listening and engagement with the thoughts or perspectives from mentors/peers was considered pivotal to cognitive and skill learning [51], personal development [53], building strong interactive relationships [55] and toward improved performance outcomes [45, 62]. Attentiveness was not merely about hearing instructions but was explained as involving an interplay of cognitive and social information exchange related to developmental or task-related problems, but which enabled individuals to fully engage with, and internalise, coaching input [61, 76]. Attentiveness was considered as being embodied in several ways, including body language (e.g. eye contact), behavioural actions (e.g. note taking; verbal clarification) or in sport via the utilisation of internal proprioceptive sensory information as well as external sources (e.g. video analysis; technical review of movement) in sport [54].

Conversely, a limited capacity to direct and regulate attention was associated with impaired coachability, primarily owing to partial or complete non-utilisation of available information. This effectively compromised the ability to respond to or accurately implement information (e.g. instruction). Distracted listening [16], mind-wandering during information availability situations [16], as well as ignoring or sporadic use of instructional feedback [4, 16] are all highlighted examples of behavioural inattentiveness, and which inhibit self-improvement [3]. Limited attentiveness has also been suggested to indicate the presence of underlying counter-responsive psychological beliefs, expectations and emotions [3], motivational resistance [16], overconfidence [16], or potentially, a lack of trust, relationship quality or respect for the coach/mentor [11, 68].

#### Willingness to Learn

Willingness to learn also emerged as a prominent constituent component, identified across 19 articles (34%) [3, 8, 11, 12, 14, 16, 19, 24, 41, 50, 51, 53, 54, 56, 58, 62, 64, 69, 76]. Willingness to learn was seen again as a dynamic component, bridging the gap between observable behaviours and the underlying thoughts and emotions driving them [3]. Willingness was often associated with an individual’s intrinsic motivation (or internal reasoning) to seek and acquire new knowledge, information and/or develop skills related to domain task/performance development [11, 12, 16]. Willingness descriptions extended beyond notions of instructional receptivity, toward an individual’s approach to learning opportunities and expressing the need for challenges for personal development [16, 58, 76]. Demonstrating a willingness to learn was intertwined with behaviours such as active intent in training/practice [3], consistent effort and intent to either self-improve or learn from mentors/instructors [58], and, by demonstration of humility, by acknowledgement of an individual’s existing skill/task limitations [8, 50].

Demonstrated high willingness was also seen as indicated by the intent and capability to reflect on learning experiences [51]. Reflection and explanation of how instructions, feedback and/or other information sources were utilised to foster personal growth and performance development, explanation and knowing of the learning/tasks required to excel in domains, and alignment with coach/mentor expectations [16, 51, 62]. If behaviours were supported by less observable but positive beliefs (e.g. efficacy and capability to develop) and emotions (e.g. low fear, anxiety), willingness was deemed internally facilitated [8, 54].

Willingness to learn was also conceptualised from the identification of behaviours, cognitions and emotions, reflecting a low willingness. These often referred to instances where behavioural or verbal resistance to feedback, learning and change was apparent. For instance, individuals may exhibit disinterest in acquiring new knowledge or skills, owing to a low perceived value or benefit (i.e. a cost–benefit approach; [19, 54, 76]). There may be negative beliefs (e.g. coach capability) or social relationships (e.g. mistrust) that may undermine willingness. Alternatively, learner overconfidence and a limited perceived need to develop or improve would also undermine willingness behaviour [69].

#### Persistence in Overcoming Setbacks

Persistence in overcoming learning setbacks was also consistently identified in seven articles (13%) as a coachability component, and deemed critical to overcoming learning and development challenges [3, 11, 13, 48, 53, 55, 57]. The literature portrayed behavioural persistence as reflecting an individual’s capability to maintain consistent effort, commitment and adaptive behaviour in response to challenges, mistakes or failures encountered during learning processes. Beswick [3] and Solomon [53] remarked on persistence as the learned capability to recognise mistakes, accept responsibility and commit to avoid repeating errors. Meanwhile, Larkin and O’Connor [55] described persistence through an individual’s ability to bounce back after setbacks, with the underlying capability of not permitting emotions or disruptive thoughts to perturb requirements to continue effort investment and learning toward tangible outcomes.

Whilst not an exhaustive list, several examples of observable behaviours indicative of high persistence were identified, including: coping with critical feedback by applying coach/mentor suggestions to the training process; maintaining composure and not becoming angry, frustrated or taking criticism personally [11, 13]; handling pressure by staying focused in high-pressure competitive situations [48]; and overcoming adversities by continuing to attempt skills and actions after making mistakes, and via demonstrating the capability to “step up and go for the next action” following setbacks [55]. By contrast, low persistence can logically be associated with low effort or effort sustainment, low engagement in problem solving, as well as task abandonment when faced with learning or performance difficulties or setbacks. These behaviours may also be likely accompanied by mal-adaptive emotions (e.g. anger, fear, frustration) and cognitive reasoning (e.g. defensive/self-protection, externalising blame, misattribution). These types of cognitive, emotional and behavioural responses were associated with hindering personal development (coach interviews).

#### Feedback Seeking

Feedback seeking emerged as a key coachability component, reported across 21 articles (40%) [13, 16, 17, 19, 23, 24, 41, 44, 45, 56–58, 64, 67–69, 71, 72, 74–76]. Often referred to as an individual’s desire and active pursuit of information for self-correction, learning and mastery [19, 57], feedback seeking was deemed important for optimised self-development, via developing the capability to seek, assimilate, integrate and utilise obtained feedback information [57]. Feedback seeking was commonly associated with observable proactive behaviours, such as regularly approaching mentors or coaches to ask clarification questions in uncertainty, for instructional guidance and for feedback following task engagement [16, 44]. Seeking feedback was also deemed to signify humility regarding an individual’s present capabilities, as well as illustrating respectful acknowledgement and gratitude for those with advanced knowledge/skills but also assisting an individual’s developmental journey [19, 20]. Relationships with other coachability components were also reported. For instance, higher frequency feedback seeking was associated with a higher willingness to learn and feedback receptivity [20, 57, 58, 67].

In business contexts, founders of ‘start-up’ companies who actively sought and integrated feedback from mentors were often evaluated as more innovative and adaptable, linking feedback-seeking behaviours with individual growth and adaptability [20, 71]. While in sport, athletes who actively sought feedback from coaches were found to enhance their skills and performance significantly relative to others [9, 45]. Iwasaki et al. [45] also identified how caring and task-involving psychological climates were likely influential to feedback-seeking behaviour, highlighting how the quality of relationships and interactions with mentors/coaches was influential. In contrast, selective, low or no feedback seeking was often seen as a barrier to coachability [57, 68], acting as a potential restraint to accessing valuable information, which otherwise would be beneficial to self-development [57, 77]. Selective feedback seeking or feedback avoidance was reported as possibly occurring for several reasons, including fear/anxiety of negative evaluations (e.g. being exposed or seen as incompetent) and ego-defensive and self-image protection reasons, or may potentially associate with a perceived psychological challenge (cognitive, emotional responses) from needing to behaviourally develop or change [23, 57].

#### Feedback Receptivity

Feedback receptivity also emerged as a prominent coachability component in 21 articles (40%) [12, 14–17, 23, 44, 47, 50, 53, 56–58, 62, 64, 67, 69–71, 73, 76]. Receptivity was commonly associated with an individual’s psychological state during learning processes, task/skill performance or in situations of evaluation. An openness to receive, accept, process, and reflect and internalise feedback (e.g. constructive criticism) without being emotionally perturbed or disrupted [12, 44, 47, 56, 67, 69, 73] or whilst maintaining emotional regulation were seen as connected characteristic features. Such receptivity enabled individuals to use feedback productively, with less inhibition and greater utilisation to aid self-learning, skill development and performance [17, 23, 44, 47, 56, 67].

Highly receptive individuals were associated with a range of observable behaviours within contexts, such as welcoming guidance and instructional advice [57], maintaining composure while correcting mistakes in response to feedback [44, 47, 73], and being trustful of the knowledge and information provided by coaches/mentors [12, 15, 50, 62, 64]. By comparison, low receptivity behaviour was characterised by dismissive, resistance or defensive behaviours when receiving advice [57, 58]. These included making excuses for not responding [23], frequent interruptions, or displaying impatience to instruction or feedback [16] as well as becoming emotionally upset [44], angry or anxious during feedback exchanges [12]. Humility was again reported as a precursor to receptivity, with a corresponding higher likelihood of interpreting feedback as valuable for growth as opposed to a personal critique of skill, performance or capability more broadly [17]. By comparison, individuals with an inflated false sense of efficacy or skill capability, who saw limited benefit from mentor or coach instruction, were more likely to be sceptical or dismissive, indicating low receptivity [17].

#### Feedback Implementation

Feedback implementation was consistently identified as a coachability component across 15 articles (28%) [3, 10–12, 23, 41, 50, 56–58, 62, 63, 67, 75, 76] and often seen as the final-action step in the feedback process. Implementation commonly referred to an individual’s capability to act or implement feedback received to adapt, modify and improve skills or behaviours [10, 50, 56] underpinning personal development [63, 67]. Despite potentially receiving similar instructional or coaching guidance, converting feedback — whether actively sought or unsolicited — into tangible outcomes was often associated as a differentiator of high and low coachability [57, 58, 63]. Highly coachable individuals were regarded as able to demonstrate implementation in a range of behaviours, such as applying feedback promptly, applying to problem solve effectively in tasks/skills, and modifying behavioural strategies or techniques [3, 10, 12, 50]. For instance, in team sports contexts where feedback can be immediate and frequent, highly coachable players were regarded as being able to make rapid in-game technical movement and tactical decision-making adjustments based on feedback [12]. Similarly in individual sports (e.g. diving), the capability to proprioceptively control and adjust movement techniques was emphasised both as a performance requirement and coachability competency for improvement (coach interviews). By comparison in business contexts, the literature highlighted how highly coachable employees integrated strategies following feedback discussions, such as task prioritisation (complete high-priority tasks first) [57, 59], enrol in specific training following feedback about a specific skill gap (e.g. presentation skills) [23, 67] and improve collaboration with others (e.g. by participating more in group discussions) [75].

Conversely, low feedback implementation is characterised by a lack of behavioural change following instruction or advice [67, 75]. In athletic settings, low coachability may be characterised as a failure to adjust or change movement, leading to repeated mistakes or stagnant performance, despite feedback provision and its acknowledgement [12, 63]. In business, employees may resist change by avoiding new behavioural strategies or sticking to familiar routines, even when feedback suggests benefits from alternative behaviours [75]. Additionally, employees lacking self-awareness may continue to make similar mistakes despite receiving corrective instruction and/or feedback, indicating a disconnect between perception and performance [67].

### Interviews: Coach Practitioner Validation of Findings

Interviews with high-performance coaches in two sport contexts validated the literature definitional understanding of coachability and identified consistent contributory coachability sub-component themes (see Sect. 2.4 for interview aims/purposes). Based on their experiences, diving coaches collectively defined coachability as: “*an ability to receive and understand instruction (cognitive), leading to a mechanical (behavioural) change, undeterred by setbacks, and driven by a willingness to learn and continually improve.*” Meanwhile, field hockey coaches referred to coachability as a state of being “*curious to discover and eager to learn, be receptive to feedback, be willing to implement the feedback, and have a resilience to keep trying.*” (see Table 2). Coaches subsequently reviewed our synthesised definition based on the literature, agreeing their statements and experience aligned with our definition.

In determining the dimensional components comprising coachability, diving coaches identified the following themes as key underpinning factors: ‘Attentiveness’; ‘Willingness to Continually Learn’; ‘Comprehend Instruction’; ‘Recognise Movement Error’, ‘Seek Feedback’, ‘Overcome Setbacks/Failure’; ‘Receptivity to Feedback’; and ‘Athlete Drive’. In parallel, and with adjusted nuance to sport-context demands, field hockey coaches identified: ‘Willingness to Learn’; ‘Seeks Feedback’; ‘Attentiveness to Technical Skills’; ‘Attentive to Tactical Awareness’; ‘Persistence’; ‘Engagement’; and ‘Implementing Feedback’. Overall, these themes demonstrated general coherence and overlap with the six consistent literature identified components. Of note, within interviews, coaches frequently provided examples of athlete cognitions and behaviours where attributes demonstrating high and low coachability were apparent, and described how these affected either athlete learning and progress or coach interactions. As such, ecological relatedness and explanatory value within sport settings aligned with research findings was achieved.

## Discussion

### Summary of Findings

The purpose of this systematic review was to (*Aim 1*) establish a consensus and a common definition of coachability and (*Aim 2*) identify constituent sub-component characteristics. Related to *Aim 1*, findings illustrated how the definitions and framing of coachability have evolved. Early definitions focused on personality traits (e.g. agreeableness, conscientiousness) [1, 11], or stable character attributes (e.g. trust and respect for authority, emotional maturity) [11, 15], while more contemporary conceptualisations have viewed coachability as a set of learned competencies that underpin learning and self-regulatory processes [41]. Irrespective of time and context, common component themes across literature definitions included reference to ‘openness in feedback’, ‘willingness to learn’, ‘ability to apply instructions’ and ‘cognitive adaptability’. In sport, literature emphasised ‘motivation to learn’, ‘inquisitiveness’ and ‘respect for coaches’. Meanwhile, business-related definitions highlighted ‘feedback seeking’, ‘receptivity’ and ‘implementation’. When synthesising definitions across domains, we therefore considered coachability as: “*an individual’s willingness and ability to seek, receive, and act upon constructive feedback to persistently foster self-development and enhance performance in a given domain*”. Aligned to sport, such a definition assumes there is a need to develop movement, technical, or cognitive skills and competencies, and further, the information or instruction required for such development is available within training and/or competitive settings.

Affixed to *Aim 2*, literature analysis suggested the presence of six consistent coachability sub-component characteristics: *Attentiveness to information*, *Willingness to learn*, *Persistence in overcoming setbacks*, *Feedback seeking*, *Feedback receptivity* and *Feedback implementation*. These components align with the interactional processes of receiving, decision making, and enacting or responding to information derived from potentially multiple sources (e.g. coaches; mentors; managers). The literature intimated the relative importance of observable behaviours indicative of high coachability. Likewise, potential barriers and behaviours within each component were identified. *Attentiveness to information* and *Willingness to learn* were identified as critical initial psycho-behavioural processes, while *Feedback Seeking*, *Receptivity* and *Implementation* were interpreted as key components in the learning and behavioural process. *Persistence in overcoming setbacks* was deemed as a key psychological and behavioural component to maintaining task investment, despite motivational, emotional, task or interactional challenges. The inter-relationships between these components suggested how the degree of engagement across component characteristics could be collectively combined to determine an individual’s overall degree of coachability.

### Research Limitations

Although coachability as a term reflecting individual attributes has prevalent colloquial use in practical sport and business contexts, numerous research limitations and investigatory gaps are evident. The theoretical framing of coachability has been inconsistent, approximately 50% of published peer-reviewed studies did not use a specific theoretical framework, and no study has critically evaluated theoretical understandings of coachability. When frameworks have been employed, like other fields, they have evolved in accordance with psychological research paradigms. Initially, understanding of problematic (low) coachability was viewed from sub-optimal trait status, clinical personality-based or underlying psycho-dynamic conflict perspectives [1, 4]. This was followed by an examination of dispositional trait approaches [43], including personality theory and the Five-Factor Model [10, 12] associations with high coachability and attainment. Subsequently, Giacobbi et al. [15, 20] framed coachability from an interactionist perspective (i.e. achievement goal theory [41, 58]), suggesting coachability may be determined by a combination of stable attributes interacting and responding with social coach/mentor exchanges. Alternative frameworks within the paradigm were also introduced (e.g. social exchange theory [19], expectancy theory [48], symbolic interactionism [50]), and have continued to some extent [19, 58, 61, 67, 69]. Most contemporary views understand coachability as a set of learned, dynamic, psychological and behavioural competencies, learned and acquired in supportive environments that match or resemble actual task/performance environments (coaching competency model [41, 84], experiential learning theory [20, 41]).

Regarding investigative attention gaps, review findings indicated that while progressive growth in sport psychology/coaching and business contexts (*n* = 40, 75% of articles) occurred, with a relatively balanced examination of male (53.5%) and female (46.5%) samples, the context of investigation was USA-concentrated (*n* = 33; 62%), with only isolated investigations conducted elsewhere. Furthermore, within US studies, populations examined primarily focused on national college athletes or coaches in the National Collegiate Athletic Association (*n* = 10; 33%) predominantly in basketball, swimming or soccer. The limited scope of geographical, cultural, demographic and contextual aspects of investigation suggests value in a broader research investigation [17]. Expanded insight could inform current definitional understanding, universal or culturally specific conceptualisation, the need for broad or context-specific nuanced assessment and how individual-cohort coachability can be evaluated.

Table 3 provides a chronological history of coachability assessment instruments utilised across domains and contexts. A review reveals that limited, yet diverse, instruments have been developed and adapted according to the investigatory domain and context. Twenty-six (49%) articles assessed coachability using an instrument listed, with particular instruments more frequently adopted. Table 3 also shows how assessment methods have been driven by theoretical underpinnings, progressing from the assessment of psychological personality dimensions to the assessment of multiple observable behavioural competencies. Initially, only descriptive singular ratings scales were evident [4], until Smith et al. [44] first conducted a peer-reviewed systematic assessment, via incorporation of a coachability subscale within their assessment of athletic coping skills (i.e. Athletic Coping Skills Inventory-28). Subsequently, Giacobbi et al. (2000, 2002) [11, 15] 24-item Athletic Coachability Scale was the first to position coachability as a distinct construct, assessing relational (e.g. coach trust, athlete collaboration) as well as individual openness and responsiveness to feedback as component features. Adaptation and assessment of additional coachability components followed (e.g. [12]), including Athletic Coachability Scale adaptations to business (e.g. sales [13]).

**Table 3:** **S**ummary of coachability associated assessment approaches derived from identified studies

Study	Questionnaire/instrument name	Construct(s) assessed and sub-scale components	Number and scoring of items	Self/observer assessed	Coachability component(s) assessed
Smith et al. (1995) [44] [Sport]	Athletic Coping Skills Inventory-28 (ACSI-28)	Assessed coping skills in athletes *Sub-scales include:* Coping with Adversity, Peaking Under Pressure, Goal-setting/Mental Preparation, Concentration, Freedom from Worry, Confidence and Achievement Motivation and Coachability	ACSI = 28-item instrument Seven subscales with 4 items per subscale 4-point Likert scale, range from 0 (almost never) to 3 (almost always)	Athlete self-reported	Coachability was one sub-scale within coping responses [Items targeted: response to coach criticism (× 2); response to coaching instruction; response to a mistake] Partial alignment with *Attentiveness*; *Persistence in Overcoming Setbacks*; *Receptivity*
Piedmont et al. (1999) [10] [Sport]	Five-Factor Personality scale (FFM) and performance relevant items	FFM assessed personality characteristics of athletes *Sub-scales include:* Openness, Conscientiousness, Extraversion, Agreeableness and Neuroticism Assessed performance-related outcomes: Coachability (1 item) Ability (1 item) Game performance (1 item) Team playerness (1 item) Work ethic (1 item)	FFM = 80-item instrument FFM = five sub-scales 7-point Likert scale, range from 1 (below average) to 7 (above average) Performance single items 7-point Likert scale, range from 1 (below average) - 7 (above average)	FFM and performance items = head and assistant coach observer reported	Several components from FFM and single item from performance assessment in alignment FFM partial alignment with *Feedback Receptivity; Attentiveness to Information; Feedback Implementation; Willingness to Learn* [Performance item targeted: player ability to listen, learn and apply coaches’ instruction] Partial alignment with *Attentiveness to Information; Willingness to Learn; Feedback Implementation*
Giacobbi et al. (2000)^a^ [11] [Sport]	Athletic Coachability Scale (ACS)	Assessed coachability in athletes *Sub-scales include:* Intensity of Effort (5 items) Reactions to Coaching Feedback (4 items), Openness to Learning (3 items), Trust/Respect for coach (4 items), Ability to Cope with Criticism (4 items), Working with teammates (4 items)	ACS = 24-item instrument Six subscales with varying number of items per sub-scale 7-point Likert scale, range from − 3 (disagree very strongly) to 3 (agree very strongly)	Athlete self-reported	Several components examined in alignment with *Willingness to Learn; Attentiveness; Persistence in Overcoming Setbacks; Feedback Receptivity*
Solomon (2008) [14] [Sport]	Solomon Expectancy Sources Scale (SESS version c)	Assessed multi-dimensional athletic ability in athletes *Sub-scales include:* Coachability (11 items) Team Player (8 items) Physical Ability (6 items) Maturity (5 items)	SESS-c = 30-item instrument Four subscales with varying number of items per sub-scale 7-point Likert scale, range from 1 (very strongly disagree) to 7 (very strongly agree)	Coach observer reported	Coachability was one sub-scale within the SESS-c [Items targeted: receptivity to coaching, willingness to listen, willingness to learn]. Partial alignment with *Attentiveness; Willingness to Learn; Feedback Receptivity*
Favor (2011) [12] [Sport]	Athlete Coachability Survey and International Personality Item Pool (IPIP)	Survey assessed athlete coachability *Survey sub-scales include:* Willingness to Listen, Learn, Change (3 items), Emotional Maturity (3 items), Determination and Commitment (2 items), Trust and Respect Coaches (3 items) Reaction to Feedback and Instruction (3 items), Positive Interactions with Others (4 items) IPIP assessed personality traits of athletes *IPIP Sub-scales include:* Emotional Stability, Anger, Anxiety, Depression, Self-Consciousness, Immoderation, Vulnerability, Agreeableness, Trust, Morality, Altruism, Cooperation, Modesty, Sympathy (5 items each)	Athlete coachability survey = 18-item instrument Six subscales with varying number of items per sub-scale 5-point Likert scale, range from 1 (almost never) to 5 (almost always) IPIP = 60-items Twelve subscales with five items per sub-scale 5-point Likert scale, range from 1 (strongly disagree) to 5 (strongly agree)	Coachability survey = coach observer reported IPIP survey = athlete self-reported	Several components examined in alignment Survey contains items aligned with *Attentiveness; Willingness to Learn; Feedback Receptivity; Persistence in Overcoming Setbacks* IPIP contains items alignment with *Persistence in Overcoming Setbacks; Feedback Receptivity*
Shannahan et al. (2013) [13] [Business]	Salesperson Coachability Scale (SCS)	SCS assessed coachability in sales employees (adapted ACS: Giacobbi et al. [15]) *Sub-scales include:* Intensity of Effort (4 items), Openness to Learning (3 items), Relationship with Coach (3 items), Coping with Feedback (8 items), Working with teammates (4 items)	SCS = 24-item instrument Five subscales utilised (as opposed to 6 in ACS) with varying number of items per sub-scale 7-point Likert scale, range from − 3 (disagree very strongly) to 3 (agree very strongly)	Salesperson self-reported	Several components examined in alignment with *Willingness to Learn; Attentiveness; Persistence in Overcoming Setbacks; Feedback Receptivity*
Ciuchta et al. (2018) [19] [Business]	Entrepreneurial Coachability Scale (ECS)	ECS assessed coachability in business entrepreneurs *Sub-scales include:* Seek-integrate (4 items), Consider (5 items)	ECS = 9-item instrument Two subscales with different number of items 5-point Likert scale, range from 1 (very strongly disagree) to 5 (very strongly agree)	Investor observer reported	[Items targeted: wants to learn, appears attentive, proactively seeks help and advice, does not get upset/angry when given feedback] Partial alignment with *Attentiveness; Willingness to Learn; Feedback Seeking; Feedback Receptivity*
Johnson et al. (2021) [17] [Business]	Workplace coachability scale (WCS) and International Personality Item Pool (IPIP) and Feedback Orientation Scale (FOS) and Learning Goal Orientation Scale (LGOS)	WCS Assessed coachability in the workplace WCS three-dimensional version *Sub-scales include:* Comfort with Coaching (3 items), Development Orientation (5 items), Acceptance of Feedback (4 items) WCS Five-dimensional version *Sub-scales include:* Comfort with Coaching (4 items), Development Orientation (5 items), Feedback Humility (6 items), Emotional Reaction to Feedback (4 items), Orientation toward Negative Feedback (5 items)	*WCS Three-dimensional* = 12-item instrument Three subscales with varying number of items per sub-scale. Scoring unknown *WCS Five-dimensional* = 24-item instrument Five subscales with varying number of items per sub-scale. Scoring unknown Manager reported: 5-point Likert scale, range from 1 (strongly disagree) to 5 (strongly agree)	*Three- and five-dimensional* Employee self-reported WCS Abbreviated 4–item instrument Manager observer reported	Several components examined in alignment *Three-dimensional* *Feedback Seeking; Willingness to Learn; Feedback Receptivity* *Five-dimensional* *Willingness to Learn; Feedback Seeking; Feedback Receptivity*
Johnson et al. (2021) [17] [Business] *continued*		IPIP (10 items) [derived from Goldberg, (1999)] Mini – IPIP (4 items) [derived from Donnellan et al., (2006)] FOS (20 items) [derived from Linderbaum and Levy (2010)] LGOS (8 items) [derived from Button et al., (1996)]	IPIP = 10 items 5-point Likert scale. range from 1 (very inaccurate) to 5 (very accurate) Mini—IPIP = 4 items 5-point Likert scale, range from 1 (very inaccurate) to 5 (very accurate) FOS = 20 items 5-point Likert scale, range from 1 (strongly disagree) to 5 (strongly agree) LGOS = 8 items 7-point Likert scale, range from 1 (strongly disagree) to 7 (strongly agree)	IPIP and Mini-IPIP survey = employee self-reported FOS survey Employee self-reported LGOS survey Employee self-reported	
Weiss and Merrigan (2021) [23] [Business]	Employee Coachability Scale (ECS) Coachee Adaptability	ECS assessed employee coachability *Sub-scales include:* Feedback Seeking Behaviours (6 items) [derived from Dahling et al., (2012)] Feedback Receptivity (6 items) [derived from Ryan et al., (2000)] Transfer of Coaching (5 items) [derived from Fateau et al., (1995)] Coachee Adaptability [derived from Alavi et al., (2014)]	ECS comprises three prior instruments utilised to examine employees’ level of coachability: Feedback seeking: 5-point Likert-type scale, range from 1 (very infrequently) to 5 (very frequently) Feedback receptivity and implementation: 5-point Likert-type scale, range from 1 (strongly disagree) to 5 (strongly agree) Coachee Adaptability = 4-item instrument	Manager observer reported	Several components examined in alignment with *Feedback Seeking; Feedback Receptivity; Feedback Implementation*
Weiss and Merrigan (2021) [23] [Business] *Continued*			5-point Likert-type scale, range from 1 (strongly disagree) to 5 (strongly agree)		
Ober et al. (2024) [76] [Business]	Coachability Situational Judgement Task (SJT)	SJT assesses coachability in workplace scenarios *Sub-scales include:* Feedback Orientation, Learning Orientation, Relationships with Coaches, Learning Initiative, Knowledge and Skill Self-awareness, and Growth Mindset Orientation WCS [adapted from ACS—Favor (2011)] *Sub-scales include:* Willingness to Listen, Learn, and Change; Emotional Maturity; Determination and Commitment; Trust and Respect for Coaches; Reaction to Feedback and Instruction; and Positive Interactions with Others	SJT = 22-item instrument SJT comprises 6 dimensions with varying items per sub-scale Single-select multiple-choice (SSMC) format WCS = 18 items Six subscales with five items per sub-scale 5-point Likert scale, range from 1 (strongly disagree) to 5 (strongly agree)	SJT = employee observer reported WCS = employee observer reported	SJT = several components examined in alignment with *Willingness to Learn; Feedback Seeking; Feedback Receptivity; Feedback Implementation; Attentiveness* WCS = survey contains items aligned with *Attentiveness; Willingness to Learn; Feedback Receptivity; Persistence in Overcoming Setbacks*
Somia et al. (2024) [41] [Education]	Behavioural Event Interview (BEI) and Coachability Survey in Entrepreneurship Education (CSEE)	BEI assesses responses to events in job roles/performance CSEE assesses entrepreneurship education coachability *Sub-scales/clusters include:* Self-Awareness (8 items), Commitment (8 items), Learning (6 items), Relationships (10 items), Implementation (8 items) *Competencies for each cluster:* SA: Self-Reflection; Self-Efficacy; Self-Assessment Com: Seeking Advice, Resilience, Achievement Orientation Learn: Receiving Feedback, Critical Reflection, Conceptualisation Rel: Building Relationships, Persuasion, Team Working Imp: Transfer learning into Action, Take the Initiative, Flexibility	BEI = 1-h semi-structed interview CSEE = 40-item instrument CSEE assesses five competency clusters and fifteen coachability competencies (3 per cluster) Competency scored first on Level of Importance on Likert scale, range from 1 (limited) to 5 (very high) Competency then scored on Level of Possession on Likert scale, range from 1 (I can’t do it) to 5 (I can do it very well)	Entrepreneur self-reported and mentor/teacher observed	Several components examined in alignment with *Feedback Seeking; Feedback Receptivity; Feedback Implementation*

*ACS* Athletic Coachability Scale, *ACSI-28* Athletic Coping Skills Inventory-28, *BEI* Behavioural Event Interview, *Com* commitment, *CSEE* Coachability Survey in Entrepreneurship Education, *ECS* Entrepreneurial Coachability Scale, *FFM* Five-Factor Model, *FOS* Feedback Orientation Scale, *Imp* implementation, *IPI* International Personality Item Pool, *Learn* learning, *LGOS* Learning Goal Orientation Scale, *Rel* relationships, *SA* self-awareness, *SESS* Solomon Expectancy Sources Scale, *SCS* Salesperson Coachability Scale, *SJT* Situational Judgement Task, *WCS* Workplace Coachability Scale

^a^Grey literature (e.g. dissertations). Table content includes assessment instruments published in peer-reviewed journals or PhD theses. Descriptive instruments or checklists within other manuscript types (e.g. book chapters) were not included

In the transfer to business settings, researchers have begun to more comprehensively assess coachability component dimensions, evident via the integration of previously validated scales (e.g. the Employee Coachability Scale [12, 14, 17, 23]) and incorporation of an independent observational assessment. Viewed through a skill competency lens, recognising that competencies are applied differently according to context, alternative assessment methods have emerged. Most recently, Ober et al. [76] observed simulated contextual scenarios and were subsequently prompted to select a course of action from a range of possible options. Responses were observer rated using the developed 22-item Coachability Situational Judgement Test, which assessed several coachability components aligned with review findings and demonstrated convergent validity with the Workplace Coachability Scale [17]. Concurrently, as part of entrepreneurship educational training, Somià et al. [41] combined mixed-method Behavioural Event Interviews with a 40-item Coachability Survey in Entrepreneurship Education. Interviews elicited a qualitative recall of specific events or situations where specific competencies were effectively implemented to achieve a result, solve a problem or manage a relationship. Afterward, the Coachability Survey in Entrepreneurship Education then assessed whether 15 developable competencies, associated with five coachability sub-component clusters, were utilised. These novel methods identify potential in more accurately assessing coachability levels in context-specific dynamic situations, employing a multiple perspective assessment.

### Future Directions

In consideration of the study findings and limitations, several prioritised future research directions can be recommended. Assuming constituent components and a synthesised definition are upheld, research will need corresponding valid and reliable assessment instruments with cross-context applicability, but with the potential for context adaptability. For enhanced assessment accuracy, development is recommended to combine self-report with external evaluation, helping mitigate potential rating bias or error common in self-report and observational methods [82, 83]. Still, observation in content, event reviewing and qualitative interview methods should also be examined for their potential to elicit novel insight [41, 45].

With assessment instrument(s), longitudinal studies will need to determine the relative (in)stability of coachability as well as identify factors and processes that undermine or facilitate coachability [76]. Identifying antecedent individual, social and environmental factors affecting coachability behaviours will be informative [67]. For instance, future research is advised to consider the influence of age, cognitive maturation and sporting experience (e.g. task familiarity) on coachability. Exploring the influence of individual antecedent factors, such as self-regulation, self-reflection and humility to coachability is warranted. These psychological characteristics may influence an individual’s likelihood to be attentive to information, engage with feedback, persist through setbacks and implement coaching instructions effectively. Previous studies do identify how pre-existing coach–athlete or leader–mentee relationships, characterised by trust, honesty, frequent feedback and open communication [8, 9, 11, 79], associate with subsequent responses. Similarly, coaches/leaders who themselves demonstrate high coachability behaviours and who create environments promoting continuous self-development and psychological safety facilitate coachability [19, 45, 77]. That said, assessment and understanding of influences from individual psychological antecedent factors (e.g. humility, self-regulation) are not established. Considered as a set of inter-individual competencies, research will need to confirm the proposed relationships with developmental outcomes, such as greater rates of learning and performance improvement. Further, determining how coachability can be optimised to achieve developmental and performance-related outcomes needs examination [17, 41, 67, 76]. Collectively, these directions could generate valuable practical knowledge and information for talent identification, selection/hiring and training development purposes in sport, business and educational contexts.

## Conclusions

This systematic review synthesised existing research available on coachability across sport, business and educational domains. With no prior consistent definition available, a synthesised definition was established, with coachability reflecting: “*an individual's willingness and ability to seek, receive, and act upon constructive feedback to persistently foster self-development and enhance performance in a given domain*”. The definition incorporates six proposed constituent sub-dimensional components, consistently identified across the literature: *Attentiveness to information*, *Willingness to learn*, *Persistence in overcoming setbacks*, *Feedback seeking*, *Feedback receptivity* and *Feedback implementation*. While receiving practitioner validation, subsequent research will need to validate the proposed sub-components. Given variability in prior conceptualisation, assessment and application, findings emphasise the need to develop standardised, multi-dimensional, coachability assessment instruments. Research should examine broader cultural contexts, sample populations and environments in which coachability may be significant to learning and performance. Further, unveiling the individual, social, relational and contextual factors affecting coachability, as well as determining whether and how coachability can be optimally developed, will be important. By advancing our understanding of coachability, researchers and practitioners could more effectively support individual growth and performance.

## Declarations

### Availability of Data and Material

This systematic review synthesised data from 53 publicly available articles (41 peer-reviewed journal articles, four book chapters and eight PhD dissertations) identified through systematic searches of databases, including CINAHL, Google Scholar, MEDLINE, PsycINFO, Scopus, SPORTDiscus and Web of Science. Details of the included studies are provided in Table 1. The full list of references is available in the References section, and the original sources can be accessed through their respective publishers or repositories, subject to access restrictions. Qualitative data from semi-structured focus-group interviews with elite diving (*n* = 5) and field hockey coaches (*n* = 3) were used to validate findings. Because of ethical considerations and confidentiality agreements with participants, the raw interview transcripts are not publicly available. The findings from these interviews are presented in an aggregated and anonymised format to protect participant privacy. For further enquiries regarding interview data, please contact the corresponding author: stephen.cobley@sydney.edu.au.

## References

[1] *Ogilvie BC, Tutko TA. Problem athletes and how to handle them. London: Pelham Books; 1966.

[2] McAdams DP, Pals JL. A new Big Five: fundamental principles for an integrative science of personality. Am Psychol. 2006;61:204–17. 10.1037/0003-066X.61.3.204. 16594837 10.1037/0003-066X.61.3.204

[3] * Beswick B. One goal. 1st ed. Human Kinetics. Champaign: Illinois; 2015.

[4] *Tutko TA, Richards JW. The psychology of coaching. Boston (MA): Allyn and Bacon; 1971.

[5] Bird B. Toward a theory of entrepreneurial competency. In: Katz JA, Brockhaus RH, editors. Advances in entrepreneurship, firm emergence, and growth. Greenwich: JAI Press; 1995. p. 251–72.

[6] Man TWY, Lau T, Chan KF. The competitiveness of small and medium enterprises: a conceptualization with focus on entrepreneurial competencies. J Bus Ventur. 2002;17:123–42. 10.1016/S0883-9026(00)00058-6.

[7] Morris MH, Webb JW, Fu J, Singhal S. A competency-based perspective on entrepreneurship education: conceptual and empirical insights. J Small Bus Manag. 2013;51:352–69. 10.1111/jsbm.12023.

[8] *Driska AP, Kamphoff C, Armentrout SM. Elite swimming coaches’ perceptions of mental toughness. Sport Psychol. 2012;26:186–206. 10.1123/tsp.26.2.186.

[9] *Smith RE, Christensen DS. Psychological skills as predictors of performance and survival in professional baseball. J Sport Exerc Psychol. 1995;17:399–415. 10.1123/jsep.17.4.399.

[10] *Piedmont RL, Hill DC, Blanco S. Predicting athletic performance using the five-factor model of personality. Pers Individ Dif. 1999;27:769–77. 10.1016/s0191-8869(98)00280-3.

[11] * Giacobbi PR. The athletic coachability scale: construct conceptualization and psychometric analyses [dissertation]. University of Tennessee; 2000.

[12] *Favor JK. The relationship between personality traits and coachability in NCAA divisions I and II female softball athletes. Int J Sports Sci Coach. 2011;6:301–14. 10.1260/1747-9541.6.2.301.

[13] *Shannahan KLJ, Bush AJ, Shannahan RJ. Are your salespeople coachable? How salesperson coachability, trait competitiveness, and transformational leadership enhance sales performance. J Acad Mark Sci. 2013;41:40–54. 10.1007/s11747-012-0302-9.

[14] *Solomon GB. The assessment of athletic ability in intercollegiate sport: instrument construction and validation. Int J Sports Sci Coach. 2008;3:513–25. 10.1260/174795408787186477.

[15] *Giacobbi PR Jr, Roper E, Whitney J, Butryn T. College coaches’ views about the development of successful athletes: a descriptive exploratory investigation. J Sport Behav. 2002;25:164–80.

[16] *Bacon TR, Spear KI. Adaptive coaching: the art and practice of a client-centered approach to performance improvement. Palo Alto: Davies-Black Publishing; 2003.

[17] *Johnson MJ, Kim KH, Colarelli SM, Boyajian M. Coachability and the development of the coachability scale. J Manag Dev. 2021;40:585–610. 10.1108/JMD-06-2020-0174.

[18] Brent KM. The influence of coachability and emotional intelligence on organizational commitment and organizational citizenship behavior [unpublished doctoral dissertation]. Beaumont: Lamar University; 2017.

[19] *Ciuchta MP, Letwin C, Stevenson R, McMahon S, Huvaj MN. Betting on the coachable entrepreneur: signaling and social exchange in entrepreneurial pitches. Entrep Theory Pract. 2018;42:860–85. 10.1177/1042258717725520.

[20] *Marvel MR, Wolfe MT, Kuratko DF. Escaping the knowledge corridor: how founder human capital and founder coachability impacts product innovation in new ventures. J Bus Ventur. 2020;35: 106060. 10.1016/j.jbusvent.2020.106060.

[21] *Mitteness CR, Sudek R, Baucus MS. Entrepreneurs as authentic transformational leaders: critical behaviors for gaining angel capital. Front Entrep Res. 2010;30:3.

[22] Mitteness C, Sudek R, Cardon MS. Angel investor characteristics that determine whether perceived passion leads to higher evaluations of funding potential. J Bus Ventur. 2012;27:592–606. 10.1016/j.jbusvent.2011.11.003.

[23] *Weiss JA, Merrigan M. Employee coachability: new insights to increase employee adaptability, performance, and promotability in organizations. Int J Evid Based Coach Mentor. 2021;19:121–36. 10.24384/kfmwab52.

[24] *Shannahan KLJ, Shannahan RJ, Bush AJ. Salesperson coachability: what it is and why it matters. J Bus Ind Mark. 2013;28:411–20. 10.1108/08858621311330254.

[25] Slack T, editor. The commercialisation of sport. Oxon: Routledge; 2004.

[26] Nagel S, Schlesinger T, Bayle E, Giauque D. Professionalisation of sport federations: a multi-level framework for analysing forms, causes and consequences. Eur Sport Manag Q. 2015;15:407–33. 10.1080/16184742.2015.1062990.

[27] De Bosscher V. The global sporting arms race: an international comparative study on sports policy factors leading to international sporting success. Meyer & Meyer Verlag: Oxford; 2008.

[28] Forgas JP, Baumeister RF, Tice DM. The psychology of self-regulation: an introductory review. In: Forgas JP, Baumeister RF, Tice DM, editors. Psychology of self-regulation. New York: Psychology Press; 2011. p. 1–17.

[29] Sarkar M, Fletcher D. Ordinary magic, extraordinary performance: psychological resilience and thriving in high achievers. Sport Exerc Perform Psychol. 2014;3:46–60. 10.1037/spy0000003.

[30] Arnold R, Fletcher D, editors. Stress, well-being, and performance in sport. Routledge: Abingdon, Oxon, Oxfordshire; 2021.

[31] Davis L, Jowett S. Coach–athlete attachment and the quality of the coach–athlete relationship: implications for athlete’s well-being. J Sports Sci. 2014;32:1454–64. 10.1080/02640414.2014.898183. 24713087 10.1080/02640414.2014.898183

[32] Rice SM, Purcell R, De Silva S, Mawren D, McGorry PD, Parker AG. The mental health of elite athletes: a narrative systematic review. Sports Med. 2016;46:1333–53. 10.1007/s40279-016-0492-2. 26896951 10.1007/s40279-016-0492-2 PMC4996886

[33] Cobley S, Baker J, Schorer J. Talent identification and development in sport: an introduction to a field of expanding research and practice. In: Baker J, Cobley S, Schorer J, editors. Talent identification and development in sport. Routledge: Abingdon, Oxon, Oxfordshire; 2020. p. 1–16.

[34] Vaeyens R, Lenoir M, Williams AM, Philippaerts RM. Talent identification and development programmes in sport: current models and future directions. Sports Med. 2008;38:703–14. 10.2165/00007256-200838090-00001. 18712939 10.2165/00007256-200838090-00001

[35] Güllich A, Cobley S. On the efficacy of talent identification and talent development programmes. In: Baker J, Cobley S, Schorer J, Wattie N, editors. Routledge handbook of talent identification and development in sport. Routledge: Abingdon, Oxon, Oxfordshire; 2017. p. 80–98.

[36] Güllich A. Selection, de-selection and progression in German football talent promotion. Eur J Sport Sci. 2014;14:530–7. 10.1080/17461391.2013.858371. 24245783 10.1080/17461391.2013.858371

[37] Beechler S, Javidan M. Leading with a global mindset. In: Javidan M, Steers RM, Hitt MA, editors. The global mindset. Emerald Group Publishing Limited: Leeds; 2007. p. 131–69.

[38] Uhl-Bien M, Arena M. Leadership for organizational adaptability: a theoretical synthesis and integrative framework. Leadersh Q. 2018;29:89–104. 10.1016/j.leaqua.2017.12.009.

[39] Burns R. Adult learner at work: the challenges of lifelong education in the new millenium. Routledge: Abingdon, Oxon, Oxfordshire; 2020.

[40] Bubb S, Earley P. Leading & managing continuing professional development: developing people, developing schools. SAGE Publications Ltd: London; 2007. 10.4135/9781446216637.

[41] *Somià T, Lechner C, Pittaway L. Assessment and development of coachability in entrepreneurship education. Int J Manag Educ. 2024;22: 100921. 10.1016/j.ijme.2023.100921.

[42] Moher D, Liberati A, Tetzlaff J, Altman DG, The PRISMA Group. Preferred reporting items for systematic reviews and meta-analyses: the PRISMA statement. PLoS Med. 2009;6: e1000097. 10.1371/journal.pmed.1000097. 21603045 PMC3090117

[43] *Ogilvie BC. Psychological consistencies within the personality of high-level competitors. JAMA. 1968;205:780–6. 10.1001/jama.1968.03140370082018. 5695287

[44] *Smith RE, Schutz RW, Smoll FL, Ptacek JT. Development and validation of a multidimensional measure of sport-specific psychological skills: the Athletic Coping Skills Inventory-28. J Sport Exerc Psychol. 1995;17:379–98. 10.1123/jsep.17.4.379.

[45] *Iwasaki S, Hogue C, Fry M. Mindful engagement mediates the relationship between motivational climate perceptions and coachability for male high school athletes. J Clin Sport Psychol. 2022;16(3):246–63. 10.1123/jcsp.2021-0012.

[46] *Bebetsos E, Antoniou P. Psychological skills of Greek badminton athletes. Percept Mot Skills. 2003;97:1289–96. 10.2466/pms.2003.97.3f.1289. 15002873 10.2466/pms.2003.97.3f.1289

[47] *Bourgeois AE, Loss R, Meyers MC, LeUnes AD. The athletic coping skills inventory: relationship with impression management and self-deception aspects of socially desirable responding. Psychol Sport Exerc. 2003;4:71–9. 10.1016/S1469-0292(01)00024-3.

[48] *Becker AJ, Solomon GB. Expectancy information and coach effectiveness in intercollegiate basketball. J Sport Psychol. 2005;17:251–66. 10.1123/tsp.19.3.251.

[49] *Shannahan KLJ. The relationship of salesperson coachability, trait competitiveness, and leadership style on salesperson performance: an interactionist perspective [dissertation]. Memphis: University of Memphis; 2006.

[50] *Navarre MJ. Male college soccer coaches’ perceptions of gender similarities and differences in coach-athlete and teammate relationships: introducing the construct of relationship-performance orientation [dissertation]. Minneapolis: University of Minnesota; 2012.

[51] *Mills A, Butt J, Maynard I, Harwood C. Identifying factors perceived to influence the development of elite youth football academy players. J Sports Sci. 2012;30(15):1593–604. 10.1080/02640414.2012.710753. 22888797 10.1080/02640414.2012.710753

[52] *Kruger A, Pienaar AE, Kemp R, Nienaber A. Sport psychological characteristics of talented 13-year old adolescents. J Psychol Afr. 2013;23(4):651–4. 10.1080/14330237.2013.10820683.

[53] *Solomon G. The influence of coach expectations on athlete development. J Sport Psychol Action. 2010;1:76–85. 10.1080/21520704.2010.528173.

[54] *Salomaa R. Expatriate coaching: factors impacting coaching success. J Glob Mobil. 2015;3(3):216–43. 10.1108/JGM-10-2014-0050.

[55] *Larkin P, O’Connor D. Talent identification and recruitment in youth soccer: recruiter’s perceptions of the key attributes for player recruitment. PLoS One. 2017;12(4): e0175716. 10.1371/journal.pone.0175716. 28419175 10.1371/journal.pone.0175716 PMC5395184

[56] *Mayer B, Dale K, Brent K. Soft skills: impact of coachability and emotional intelligence on organizational citizenship behaviors. J Appl Bus Res. 2018;34(5):833–44. 10.19030/jabr.v34i5.10204.

[57] *Weiss JA. An examination of employee coachability and managerial coaching in organizations [dissertation]. Chicago: DePaul University; 2019.

[58] *Kuratko DF, Neubert E, Marvel MR. Insights on the mentorship and coachability of entrepreneurs. Bus Horiz. 2021;64(2):199–213. 10.1016/j.bushor.2020.11.001.

[59] *Wu CH, Nien JT, Lin CY, Nien YH, Kuan G, Wu TY, et al. Relationship between mindfulness, psychological skills, and mental toughness in college athletes. Int J Environ Res Public Health. 2021;18(13):6802. 10.3390/ijerph18136802. 34202770 10.3390/ijerph18136802 PMC8297292

[60] *Maxheimer MM, Nicholls-Nixon CL. What women want (and need) from coaching relationships during business incubation. J Small Bus Entrep. 2022;34(5):548–77. 10.1080/08276331.2021.1981728.

[61] *Nguyen K, Coplan RJ, Archbell KA, Rose-Krasnor L. Coaches’ beliefs about shy children and adolescents in the context of team sports. Int J Environ Res Public Health. 2021;18(12):6802. 10.3390/ijerph18136802. 34202770 10.3390/ijerph18136802 PMC8297292

[62] *Davis EL. A model of coachability: coach and athlete perspectives [dissertation]. Tucson: University of Arizona Global Campus; 2021.

[63] *Kraska J. Personality predictors of athletic performance in collegiate athletes [dissertation]. Thousand Oaks: California Lutheran University; 2021.

[64] * Somià T. Understanding coachability and its relevance to entrepreneurship education. In: Morris MH, Liguori E, editors. Annals of entrepreneurship education and pedagogy: 2023. Cheltenham: Edward Elgar Publishing; 2022: p. 165–83. 10.4337/9781803926193.00018.

[65] *Bar-On R, Fiedeldey-Van DC. The Bar-On model and multifactor measure of human performance: validation and application. Front Psychol. 2022;13: 872360. 10.3389/fpsyg.2022.872360. 35859841 10.3389/fpsyg.2022.872360 PMC9291401

[66] *Azim MG, Rahayuni K, Widiawati P. Psychological skills of Malang State University athletes who participate in POMPROV 2022. J Phys Educ Sport. 2023;23(1):123–30. 10.7752/jpes.2023.01016.

[67] *Weiss JA, Outland N, Plummer G, Zervos L, Carmichael-Tanaka N, Kang B. The stable individual differences driving employee coachability behaviours. Int J Evid Based Coach Mentor. 2023;21(2):102–17. 10.24384/d24j-fh23.

[68] *Tetteh A, Weng Q, Sungu LJ, Adams MZ. How much conflict is too much? How frequent task conflict expressions affect angels’ reinvestment intention. Int J Manag Educ. 2023;21(2):102–17. 10.24384/d24j-fh23.

[69] *James-Boone TC. Competitive advantage or non-starter: coachability from the perspective of experienced professional coaches [dissertation]. Philadelphia: Temple University; 2023.

[70] *Jooste J, Kruger A, Tinkler N. The influence of emotional intelligence on coping ability in senior female field-hockey players in South Africa. J Hum Kinet. 2023;88:123–35. 10.2478/hukin-2023-0012. 10.5114/jhk/161550 PMC10203831 37229407

[71] *Svetek M. The role of entrepreneurs’ perceived competence and cooperativeness in early-stage financing. Entrep Theory Pract. 2023. 10.1177/10422587211012345.

[72] *Letwin C, Ciuchta MP, Stevenson R, McMahon S. Being coachable can pay off for founders: up to a point. Entrep Theory Pract. 2023;47(6):2053–76. 10.1177/10422587231183930.

[73] *Kemarat S, Theanthong A, Yeemin W, Suwankan S, Makaje N. Influence of mental skills on competitive anxiety in professional futsal players: difference by successful and unsuccessful players. Curr Psychol. 2023. 10.1007/s12144-023-05159-0.

[74] *Champatiray MY, Hota SL, Kumar A, Kumar A. The sustainability of emotional intelligence and decision-making flair by financial investors through the mediating role of coachability. Folia Oeconomica Stetinensia. 2023;23(2):102–15. 10.2478/foli-2023-0021.

[75] *Fournet AC. Coaching isn’t just for little league anymore: a theory of individual coachability [dissertation]. Ruston: Louisiana Tech University; 2023.

[76] *Ober TM, Williams KM, Kell HJ, Holtzman S. Measuring coachability by situational judgment task: development and initial validation. Pers Individ Dif. 2024;214: 112503. 10.1016/j.paid.2023.112503.

[77] Callow N, Smith MJ, Hardy L, Arthur CA, Hardy J. Measurement of transformational leadership and its relationship with team cohesion and performance level. J Appl Sport Psychol. 2009;21(4):395–412. 10.1080/10413200903204754.

[78] Deci EL, Ryan RM. The “what” and “why” of goal pursuits: human needs and the self-determination of behavior. Psychol Inq. 2000;11(4):227–68. 10.1207/S15327965PLI1104_01.

[79] Shanmuganathan-Felton V, Felton L, Jowett S. It takes two: the importance of the coach-athlete relationship. Front Young Minds. 2022;10: 676115. 10.3389/frym.2022.676115.

[80] McAdams DP. The five-factor model in personality: a critical appraisal. J Pers. 1992;60(2):329–61. 10.1111/j.1467-6494.1992.tb00973.x. 1635046 10.1111/j.1467-6494.1992.tb00976.x

[81] Boyle GJ. Critique of the five-factor model of personality. In: Boyle GJ, Matthews G, Saklofske DH, editors. The SAGE handbook of personality theory and assessment, vol. 1. London: SAGE Publications Ltd; 2008. p. 295–312. 10.4135/9781849200462.n14.

[82] Hoyt WT. Rater bias in psychological research: when is it a problem and what can we do about it? Psychol Methods. 2000;5(1):64–86. 10.1037/1082-989X.5.1.64. 10937323 10.1037/1082-989x.5.1.64

[83] Podsakoff PM, MacKenzie SB, Podsakoff NP. Sources of method bias in social science research and recommendations on how to control it. Annu Rev Psychol. 2012;63:539–69. 10.1146/annurev-psych-120710-100452. 21838546 10.1146/annurev-psych-120710-100452

[84] Wise D, Hammack M. Leadership coaching: coaching competencies and best practices. J Sch Leadersh. 2011;21(3):449–77. 10.1177/105268461102100.

